# Identification of novel autophagic *Radix Polygalae* fraction by cell membrane chromatography and UHPLC-(Q)TOF-MS for degradation of neurodegenerative disease proteins

**DOI:** 10.1038/srep17199

**Published:** 2015-11-24

**Authors:** An-Guo Wu, Vincent Kam-Wai Wong, Wu Zeng, Liang Liu, Betty Yuen-Kwan Law

**Affiliations:** 1 State Key Laboratory of Quality Research in Chinese Medicine, Macau University of Science and Technology, Macau, China

## Abstract

With its traditional use in relieving insomnia and anxiety, our previous study has identified onjisaponin B from *Radix Polygalae* (*RP*), as a novel autophagic enhancer with potential neuroprotective effects. In current study, we have further identified a novel active fraction from *RP*, contains 17 major triterpenoid saponins including the onjisaponin B, by the combinational use of cell membrane chromatography (CMC) and ultra-performance liquid chromatography coupled to (quadrupole) time-of-flight mass spectrometry {UHPLC-(Q)TOF-MS}. By exhibiting more potent autophagic effect in cells, the active fraction enhances the clearance of mutant huntingtin, and reduces protein level and aggregation of α-synuclein in a higher extent when compared with onjisaponin B. Here, we have reported for the first time the new application of cell-based CMC and UHPLC-(Q)TOF-MS analysis in identifying new autophagy inducers with neuroprotective effects from Chinese medicinal herb. This result has provided novel insights into the possible pharmacological actions of the active components present in the newly identified active fraction of *RP*, which may help to improve the efficacy of the traditional way of prescribing *RP*, and also provide new standard for the quality control of decoction of *RP* or its medicinal products in the future.

Neurodegenerative diseases such as Alzheimer’s disease (AD), Parkinson’s disease (PD) or Huntington’s disease (HD), are caused by the formation of inclusion bodies and protein aggregates, or the deposition of abnormal proteins in neuronal cells, which finally lead to degeneration and selective neuronal vulnerability in specific brain regions^1^,^2^,^3^. As neurons cannot reproduce or replace themselves when they were damaged or died, progressive degeneration of structures and functions of neurons will cause problems in both physical movement (ataxias) and mental functions (dementias)^4^. Recent researches have revealed the increased formation of autophagic vacuoles in the dopaminergic neurons of PD model^5^, which suggested the possible correlation between autophagy and neurodegenerative diseases. In fact, autophagy is a catabolic mechanism which involves the degradation of dysfunctional cellular components through the autophagy-lysosomal pathway^6^. It is activated upon cellular stressful conditions such as depletion of nutrients and growth factors, hypoxia or radiation^7^. The degraded cellular components are then recycled to promote cellular survival through maintaining normal energy level in cells^8^.

*Radix Polygalae* (*RP*) (Yuan Zhi) is a common Chinese herbal medicinal plant prescribed for treatment of forgetfulness^9^, anxiety^10^, insomnia and depression^11^ in the Chinese community. The major chemical components of *RP* include saponins, xanthones, oligosaccharide esters and alkaloids^12^,^13^,^14^,^15^,^16^,^17^,^18^,^19^. Recent pharmacological studies have reported that *RP* has the sedative-hypnotic^10^, memory improving^9^, cognitive-enhancing^20^ and neuroprotective effects^19^,^21^,^22^. Moreover, *RP* activates the N-methyl-D-aspartate (NMDA) or inhibits the phosphatidylinositol 3-kinase (PI3K)/Akt signaling pathways^22^,^23^. In fact, *RP* is usually prescribed as decoctions such as “Kai Xin San” and “Ding Zhi Xiao Wan” in traditional Chinese medicine^24^,^25^, this prompts us to investigate the pharmacological and mechanistic actions of *RP*. Our previous study has suggested that onjisaponin B isolated from *RP*, induces autophagy and attenuates the protein level of mutant proteins including α-synuclein and huntingtin, which are highly associated with HD and PD respectively^21^.

In our current study, we reported that with an equal amount of onjisaponin B presents in the total ethanol extract (TEE) of *RP*, *RP* (TEE) showed more potent autophagic effect when compared with onjisaponin B alone. Based on this observation, we postulated that additional components in *RP* (TEE) may be responsible for inducing autophagy or enhancing the autophagic effect of onjisaponin B. Modern pharmacological studies have reported that compounds exert their biological effects by direct binding with receptors on the cell membrane^26^,^27^. In fact, cell membrane chromatography (CMC) method was previously used for the identification of bioactive components. For example, the human epidermal squamous cells (A431 cells) and human embryonic kidney (HEK 293 cells) coupled CMC model were used for screening of epidermal growth factor receptor (EGFRs) antagonists^28^,^29^, and the human umbilical vein endothelial cell (HUVEC) coupled CMC model was applied for analyzing the competitive binding activity on the receptor of AGEs (RAGE)^30^. To this end, we applied the CMC, ultra-performance liquid chromatography time-of-flight mass spectrometry (UHPLC-TOF-MS) and ultra-performance liquid chromatography quadrupole time-of-flight mass spectrometry (UHPLC-Q-TOF-MS) to identify the active fraction and components of *RP*, which are responsible for the autophagic and neuroprotective effects in PC-12 cells^28^,^29^,^30^. Firstly, by applying the 70 to 80% of methanol gradient system with the octadecylsilane (ODS) column, we have isolated successfully the active methanol fraction (MF) of *RP* that binds to cellular membrane of PC-12 cells as revealed by CMC. Our UHPLC-(Q)TOF-MS results further demonstrated that 17 major triterpenoid saponins, including onjisaponin B, are presented in the *RP* fraction eluted by using 70 to 80% of methanol (70–80% MF). With a more potent autophagic and neuroprotective effect induced by the active methanol fraction of *RP* (70–80% MF) when compared with onjisaponin B, the identification of the active fraction may help to further explain the pharmacological and mechanistic action of *RP*, improve the efficacy of the traditional way of prescribing *RP* decoction as medication, and also serve as a new standard for the quality control of *RP*.

## Results

### Identification of bioactive fraction from *RP* by cell membrane chromatography

*RP* is classified as a top grade herbal plant in Chinese herbal medicine (CHM). It is the main effective herb of many traditional herbal decoctions such as “Kai Xin San”, “Yuan Zhi Wan” and “Ding Zhi Wan”, which are prescribed for modulation of emotion or longevity in CHM. Although recent research findings have reported that *RP* has protective effects in neurodegenerative diseases such as improving cognitive recognition, promoting the degradation of aggregated-proteins, and antidepressant^20^,^21^,^31^, the active components responsible for the pharmacological actions of *RP* remain unclear.

**Figure 1:**
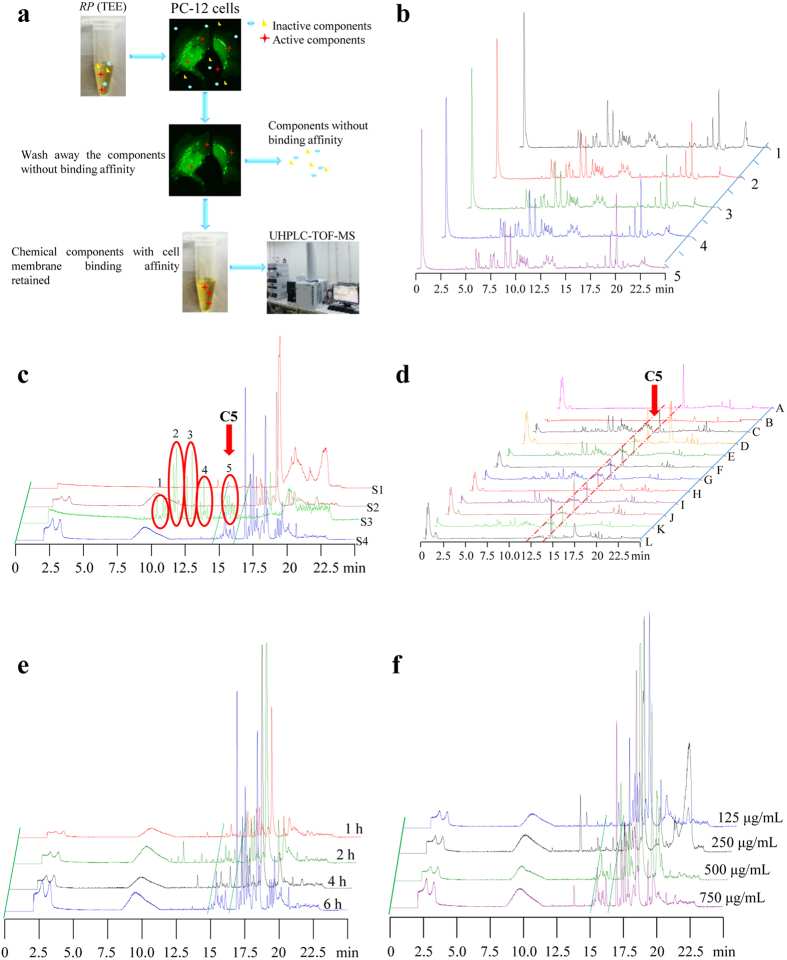
The identification of the active binding fraction of *RP* by CMC. (**a**) The experimental flow of CMC. PC-12 cells were incubated with *RP* (TEE) for 1 to 6 h. After incubation, chemical components without binding affinity to the cell membrane were washed away by PBS, while those components that bind on cell membrane were retained for analysis. The cells were then disrupted by citric acid buffer with ultrasound sonication. The lysate solution was then centrifuged, dried and re-dissolved in methanol. Cell lysate without *RP* incubation was collected as control. Finally, all the collected samples were analyzed using UHPLC-TOF-MS. (**b**) The Total Ion Chromatogram (TIC) of the 5 different batches of *RP* (TEE). (**c**) The TIC of the CMC samples. S1: The final PBS wash solution; S2: PC-12 lysate solution without *RP* (TEE) treatments; S3: *RP* (TEE) solution diluted with PBS; S4: PC-12 cell lysate solution collected after *RP* (TEE) treatments. The cluster of peaks (C5) indicated the chemical components that bind on the cell membrane of PC-12 cells. (**d**) The TIC of the CMC samples collected from 5 different batches of *RP* (TEE) treatments. A: PC-12 lysate solution without *RP* (TEE) treatments; B: The final PBS wash solution; C, E, G, I, K: 5 different batches of *RP* (TEE) solution diluted with PBS; D, F, H, J, L: PC-12 cell lysate solution collected after treatments of 5 different batches of *RP* (TEE). (**e**) The TIC of PC-12 cell lysate collected after 1 to 6 h of *RP* (TEE) treatments (500 μg/mL). (**f**) The TIC of PC-12 cell lysate collected after *RP* (TEE) treatments (125, 250, 500 or 750 μg/mL) for 4 h. All the samples were analyzed by UHPLC-TOF-MS on an Agilent Zorbax Eclipse Plus C-18 50 mm × 2.1 mm column (particle size: 1.8 μm) at a flow rate of 0.35 mL/min. The data was acquired in the scan mode from *m/z* 100 to 3200 Da with 2.0 spectra/s.

In this study, it is reported for the first time the use of PC-12 cells coupled CMC model to identify active autophagic CHM components which bind on the cell membrane (Fig. 1a). To begin, CMC was performed by incubating the *RP* (TEE) with PC-12 cells. While compounds without binding affinity to the cells were washed away, cell lysates containing compounds that bind on cell membranes were collected and analyzed by high sensitive UHPLC-TOF-MS.

The total ion chromatography (TIC) of *RP* (TEE) in negative ion pattern was performed. Under optimized chromatographic condition, 5 different batches of *RP* (TEE) were analyzed by UHPLC-TOF-MS, and all samples showed similar chromatographic peaks (Fig. 1b) which confirmed the quality of *RP* (TEE) between different batches. As shown in Fig. 1c, the chromatogram of one batch of *RP* (TEE) was divided into 5 main clusters of peaks (C1–C5) (S3), however, only C5 was detected in the PC-12 cell lysate with *RP* (TEE) incubation (S4). Consistently, C5 was not detected in the control cell lysate without treatment of *RP* (TEE) (S2), or the final PBS wash buffer residue solution (S1). This data suggested that the chemical components in C5 bind to the cell membrane of PC-12. Furthermore, CMC was also performed on the remaining 4 batches of *RP* (TEE). Consistently, C5 peaks were detected in the PC-12 cell lysate treated with different batches of *RP* (TEE) (Fig. 1d) (D, F, H, J, and L). Furthermore, PC-12 cells incubated with *RP* (TEE) at different time (Fig. 1e) and concentrations (Fig. 1f) were also investigated. The results indicated the binding efficiency of the chemical components of C5 to the cell membrane increased in a time- and dose- dependent manner.

### Identification of the chemical components in the CMC-isolated fraction of *RP* by using UHPLC-TOF-MS and UHPLC-Q-TOF-MS

**Figure 2:**
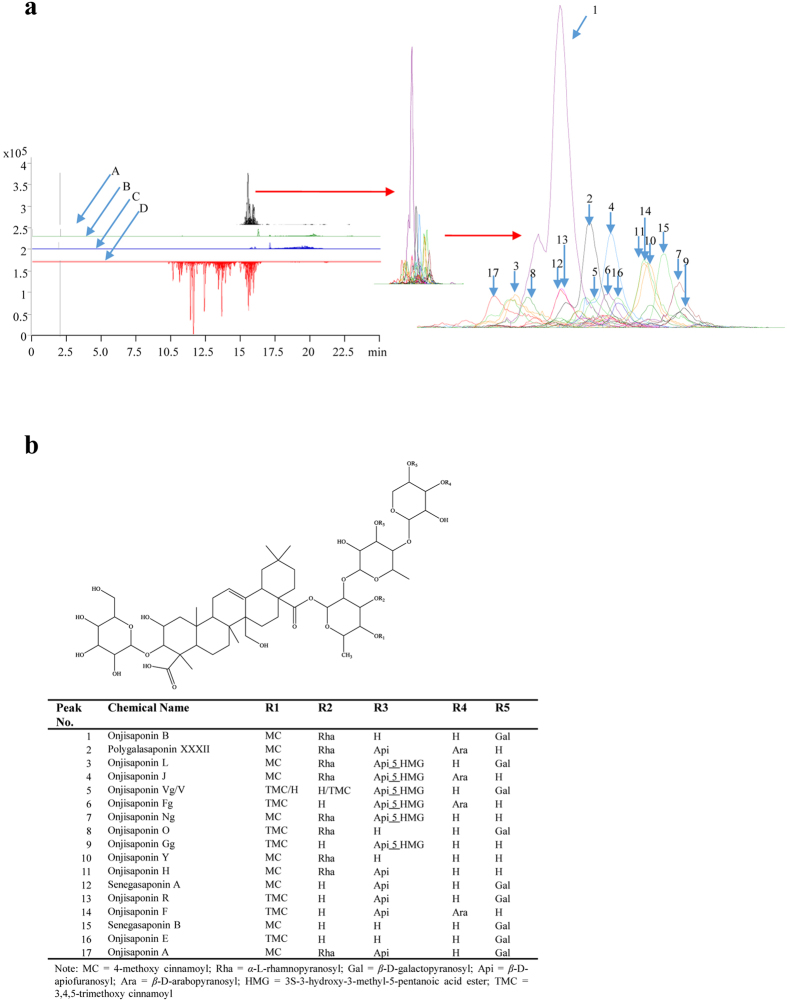
Identification of the active binding compounds of *RP* fraction isolated by CMC analysis. 17 major compounds present in the active binding fraction identified by CMC and UHPLC-(Q)TOF-MS analysis. (**a**) EIC graph showed the elution and identification of the 17 main compounds from *RP* (TEE). A: Lysate of PC-12 cells treated with *RP* (TEE); B: The final PBS wash control; C: Lysate of PC-12 cells without *RP* (TEE) treatment; D: The *RP* (TEE) diluted with PBS. (**b**) The chemical structures and names of the 17 major compounds identified from *RP* (TEE) using UHPLC-(Q)TOF-MS.

Although HPLC-MS or HPLC-UV is commonly used for chemical analysis, it is not sensitive enough to confirm the mass of the active compounds accurately in decimal places^28^,^29^,^30^. To improve the limitations of current detection methods, high sensitivity UHPLC-TOF-MS, which can accurately measure the mass of the compounds in 4 decimal places, was applied to analyze the CMC-identified fraction of *RP*. To begin, analysis on the fraction of *RP* (C5) isolated by CMC was performed by using high sensitive UHPLC-TOF-MS in the scan mode (m/z 100 to 3200 Da with 2.0 spectra/s). In the accurate mass ranging from 100 to 3200 Da, 17 major peaks were found in C5 peak (Fig. 2a). We then matched these 17 peaks with the accurate mass (MS) and the molecular formula of known compounds isolated from *Polygala* according to reported literature values^12^,^13^,^14^,^15^,^16^,^17^,^18^,^19^,^32^,^33^,^34^,^35^ and the “Dictionary of Natural Products”^36^. Finally, the chemical components present in peak 1–4, 6–11 and 15–17 of C5 were identified (Table 1 and Fig. 2b). Among them, peak 1, which is the highest abundance in C5, was confirmed as onjisaponin B (Table 1 and Fig. 2b). To improve the accuracy of the data, all these peaks were further analyzed by using UHPLC-Q-TOF-MS (Supplementary Table 1 and Supplementary Figure 1). As the chemical components present in peak 5, 12–14 have the same accurate MS and molecular formula as some other components, their identity were further confirmed by analyzing their different major fragment ions using UHPLC-Q-TOF-MS. As showed in Supplementary Table 1 and Supplementary Figure 1, 567.1976, 1155.5581, and 1125.5491 are the characteristic fragment ions for the peak 12, 13 and 14, respectively. Therefore, their identities were confirmed as senegasaponin A, onjisaponin R and onjisaponin F respectively, but not polygalasaponin XLIII, polygalasaponin XLI and polygalasaponin XXX. Peak 5 was identified as onjisaponin Vg or onjisaponin V as they share the same molecular formula (C_82_H_122_O_41_), and have the same characteristic fragment ions due to the same sugar residues^19^.

### The isolation, purification and quantitation of the fraction of *RP* (C5)

**Figure 3:**
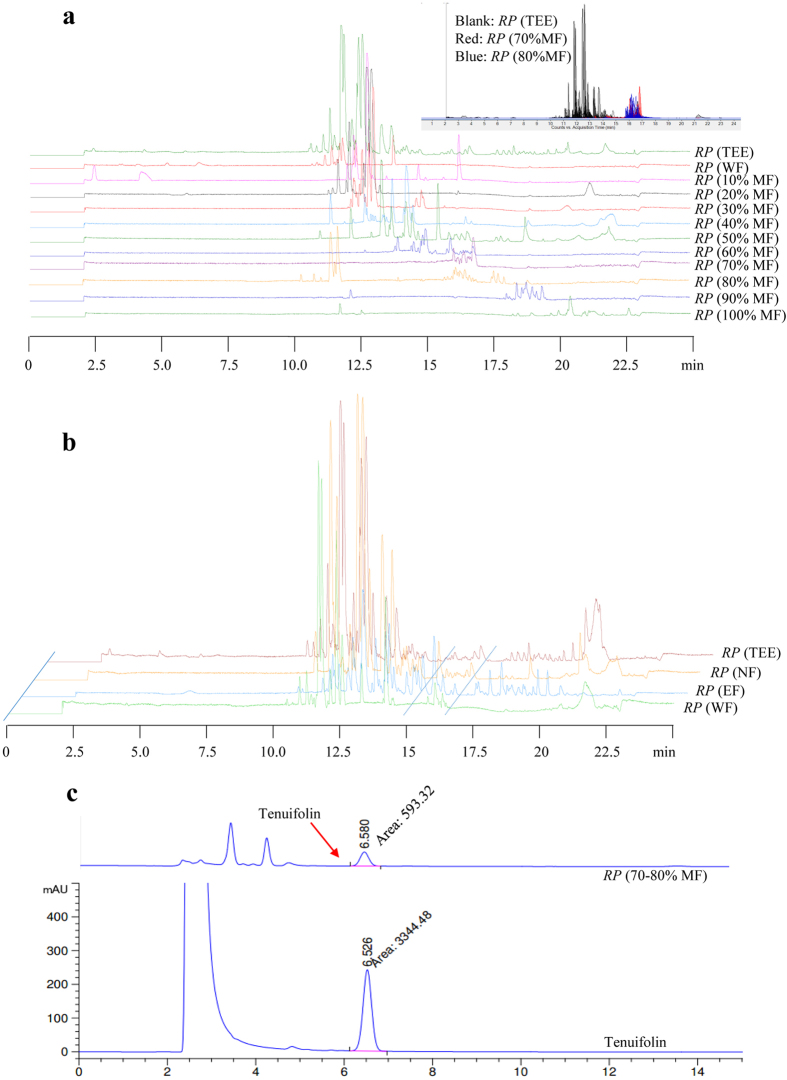
The TIC of the active binding fraction of *RP* obtained by 2 extraction methods. (**a**) Method 1: *RP* (TEE) was eluted through ODS column using water and 10% to 100% of methanol. (**b**) Method 2: *RP* (TEE) was dissolved with water and then extracted by ethylethanoate and n-Butanol sequentially. The active binding fraction (C5) of *RP* isolated by CMC was mainly presented in the n-Butanol (NF) fraction. (**c**) The quantification of the total amount of saponins present in the active binding fraction (C5) of *RP* by using tenuifolin as the reference standard.

To confirm whether the fraction of *RP* (C5) identified by CMC is responsible for the autophagic effect of *RP* (TEE), we isolated C5 by using ODS open column chromatography. Through eluting different fractions of chemical components from *RP* (TEE) by increasing the priority of the solvent system (10 to 100% of methanol), 11 fractions were collected and analyzed by UHPLC-TOF-MS. As showed in Fig. 3a, the 17 identified chemical components of C5, which have the binding affinity to cell membrane of PC-12, were eluted by 70 to 80% of methanol. By extracting *RP* (TEE) with an alternate method using ethylethanoate and n-Butanol, Fig. 3b showed that the chemical components of C5 were presented in the n-Butanol fraction. Although both methods were able to extract the fraction of *RP* (C5) successfully, there were a lot of interference peaks in the n-Butanol fraction (Fig. 3b). The results suggested that the methanol gradient solvent system using ODS column could isolate the active C5 fraction better than the n-butanol extraction system.

Furthermore, as all the 17 identified compounds present in the C5 fraction possess the same nucleus structure as the saponin reference standard (tenuifolin), therefore, we quantitated the fraction of *RP* (C5) by using tenuifolin as the reference standard according to the protocol stated in “Chinese Pharmacopoeia 2010”^37^ . As shown in Fig. 3c, the total amount of saponins present in the fraction of *RP* (C5), which is eluted by the 70 to 80% of methanol, is 86.43%. Our result on the quantitation of the total percentage of saponins present in C5, therefore, may act as an important reference standard for the quality control of related *RP* medical products currently available in the market.

### Cytotoxicity of the different isolated fractions of *RP*

**Figure 4:**
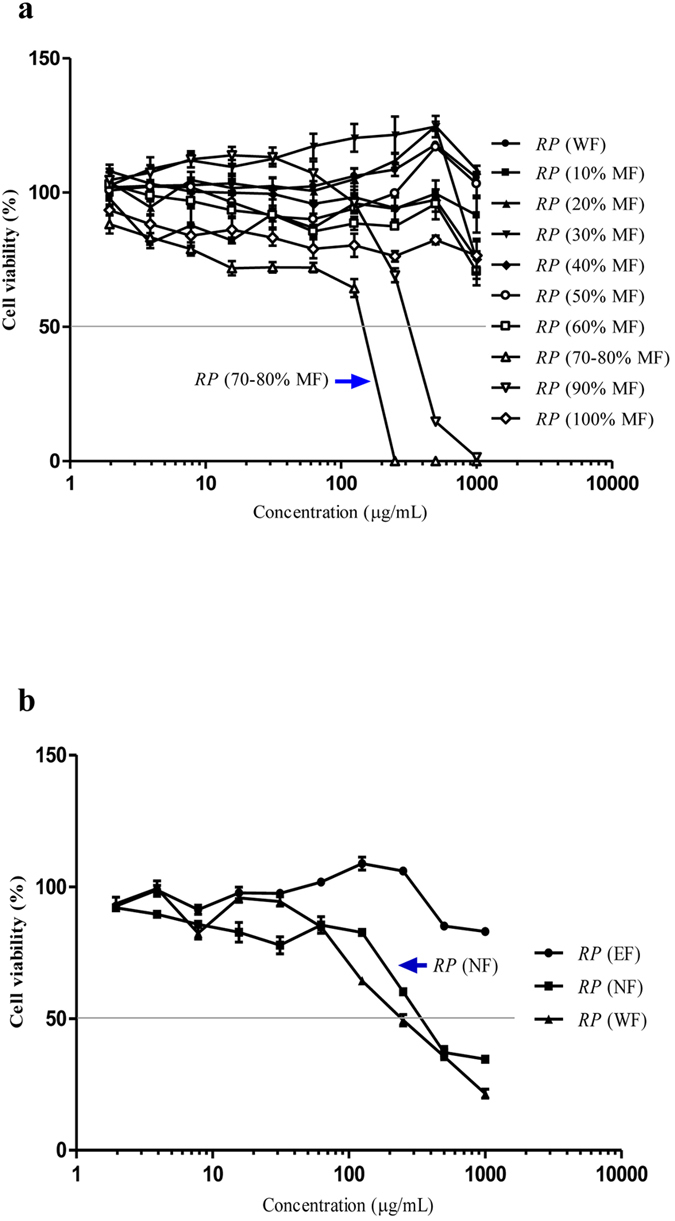
Cytotoxicity of the isolated fractions of *RP* after 48 h of treatments in PC-12 cells. (**a**) Cytotoxicity of the fractions of *RP* eluted by ODS column with water or 10% to 100% of methanol. (**b**) Cytotoxicity of the water, n-butanol and ethylethanoate fractions of *RP*.

As mentioned in the previous section, we isolated the fraction of *RP* (C5) by using (i) the gradient solvent system with 10% to 100% of methanol, (ii) ethylethanoate and n-Butanol system, respectively. To further evaluate the cytotoxic effect of all the above isolated fractions of *RP*, we measured their cytotoxicity (IC_50_ value) by performing the MTT assay. The IC_50_ value of the methanol fraction of *RP* (70–80% MF), and the n-butanol fractions (NF) of *RP*, which both contain the fraction of *RP* (C5), were determined as 144 and 338 μg/mL respectively (Fig. 4a,b). The IC_50_ results further suggested the NF of *RP* contains extra compounds which may affect the purity of the identified fraction of *RP* (C5).

### The autophagic effect of the isolated methanol fraction (70–80% MF) of *RP*

**Figure 5:**
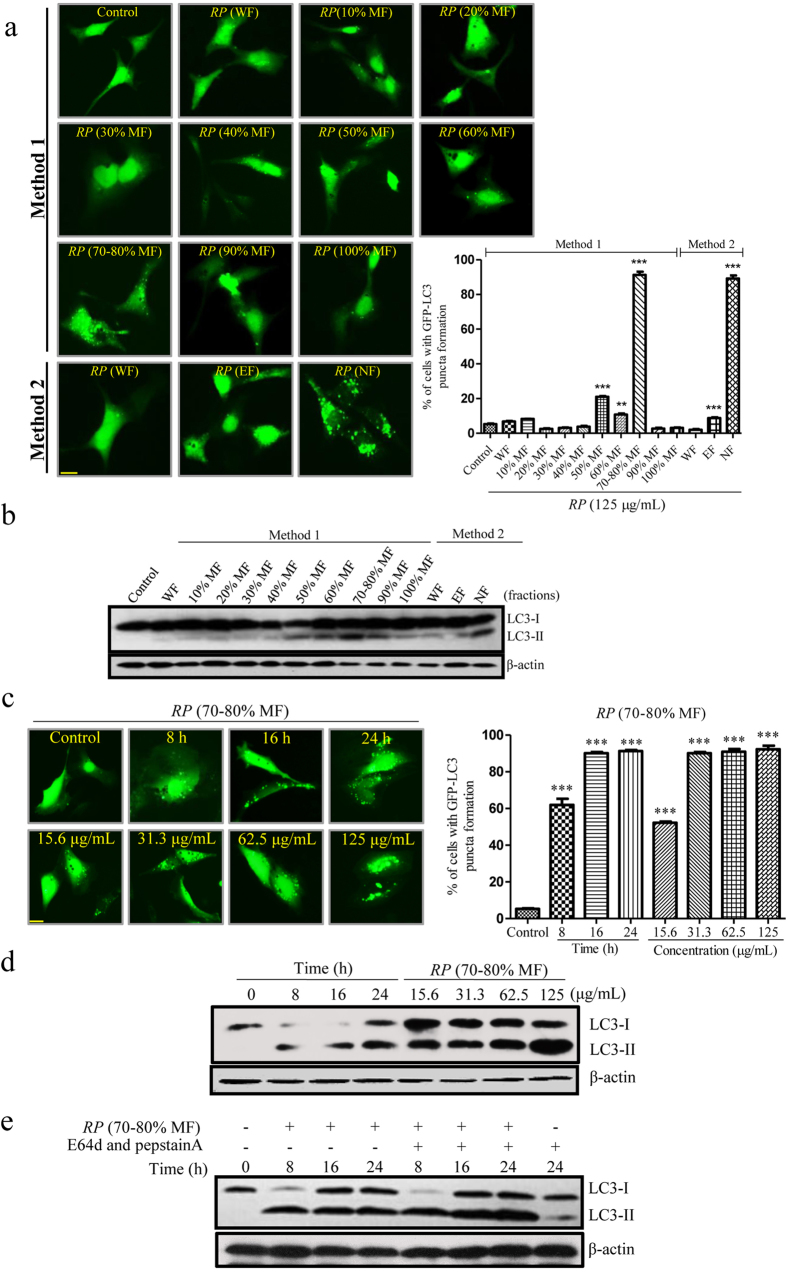
The autophagic effect of the isolated fractions of *RP* in PC-12 cells. (**a**) PC-12 cells transfected with GFP-LC3 plasmids were incubated with different fractions of *RP* (TEE) (125 μg/mL) eluted with water or 10% to 100% of methanol, or extracted by ethylethanoate and n-Butanol sequentially. Representative images showed the formation of GFP-LC3 puncta after treatments for 24 h. Right: bar chart indicated the percentage of cells with GFP-LC3 puncta formation. (**b**) PC-12 cells were treated with different fractions of *RP* (125 μg/mL) for 24 h. Cell lysates were then harvested and analyzed for LC3 I/II and β-actin, respectively. (**c**) *RP* (70–80% MF) activated autophagy in PC-12 cells. PC-12 cells transfected with GFP-LC3 plasmids were incubated with *RP* (70–80% MF) with the indicated concentrations and time. Representative images of cells showed GFP-LC3 puncta formation after treatments. Right: bar chart indicated the percentage of cells with GFP-LC3 puncta formation; (**d**) PC-12 cells were treated with *RP* (70–80% MF) at the indicated time and concentrations. Cell lysates were then harvested and analyzed for LC3 I/II and β-actin, respectively. (**e**) PC-12 cells were treated with *RP* (70–80% MF) (62.5 μg/mL) with or without the presence of lysosomal protease inhibitors (10 μg/mL) for 24 h. Cell lysates were then harvested and analyzed for LC3 I/II and β-actin, respectively. Bars, S.D. ***p < 0.001; **p < 0.01. Magnification, ×40; Scale bar: 15 μm. The full-length blots are presented in Supplementary Figure 4.

To evaluate the autophagic effect of all the isolated *RP* fractions in PC-12 cells, we first monitored the conversion of microtubule associated protein light chain 3 (LC3)-I (cytosolic) to LC3-II (membrane-bound), which is essential for the induction of autophagy, by using immunofluorescence microscopy. This was performed by expressing PC-12 cells with green fluorescent protein (GFP)-LC3 plasmids, the cells were then treated with different fractions of *RP* respectively. As shown in Fig. 5a, both the *RP* (70–80% MF) and *RP* (NF) increased the number of fluorescent LC3 II puncta in cells. Besides, immunoblotting results confirmed that the 2 fractions increased the protein level of LC3-II in cells (Fig. 5b).

Furthermore, we evaluated the autophagic effect of the *RP* (70–80% MF) with different concentrations (15.63–125 μg/mL) and treatment time (0–24 h). As shown in Fig. 5c,d, *RP* (70–80% MF) induced autophagy in a dose- and time-dependent manner. However, an increase in the formation of fluorescent LC3 II puncta formation can be caused by either the induction of autophagic flux, or the failure in the removal of autophagosomes due to the blockage of fusion of autophagosomes and lysosomes. To differentiate between these 2 possibilities, the protein level of LC3-II was evaluated in the presence of E64d and pepstatin A (lysosomal protease inhibitors). As shown in Fig. 5e, *RP* (70–80% MF) increased the formation rate of LC3-II in the presence of E64d and pepstatin A. The results therefore suggested *RP* (70–80% MF) induced autophagic activity through increased autophagosomes formation.

### Comparison of the autophagic effect of *RP* (TEE), *RP* (70–80% MF) and *RP* (NF) with onjisaponin B

**Figure 6:**
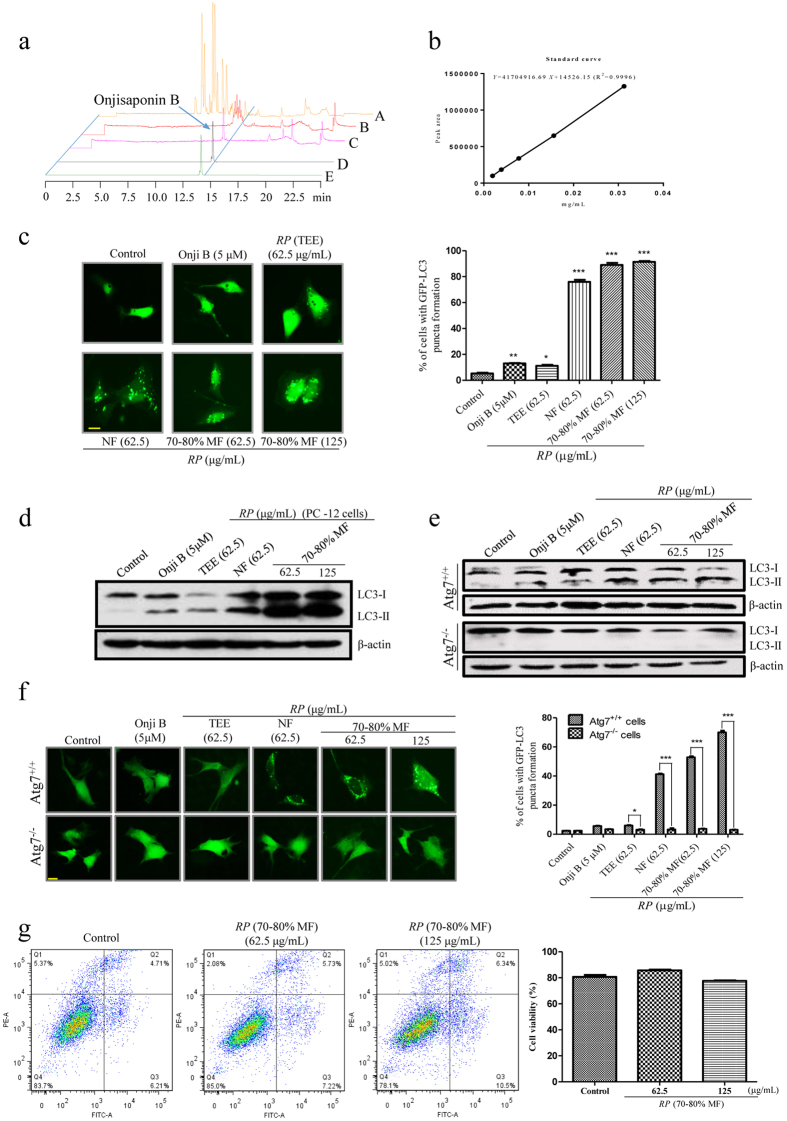
The comparison of the autophagic effect among onjisaponin B, *RP* (TEE), *RP* (NF) and *RP* (70–80% MF). (**a**) The TIC and Extracted Ion Chromatogram (EIC) of different fractions of *RP* and onjisaponin B. A: The TIC of *RP* (NF); B: The TIC of *RP* (70–80% MF); C: The TIC of onjisaponin B; D: The EIC of onjisaponin B in *RP* (70–80% MF); E: The EIC of onjisaponin B. (**b**) The standard curve of onjisaponin B. The concentration of onjisaponin B in 125 μg/mL of *RP* (70–80% MF) and *RP* (NF) are 8.89 μM and 3.23 μM, respectively. (**c**) PC-12 cells transfected with GFP-LC3 plasmids were incubated with onjisaponin B (5 μM), *RP* (TEE) (62.5 μg/mL), *RP* (NF) (62.5 μg/mL) and *RP* (70–80% MF) (62.5 and 125 μg/mL) for 24 h. Representative images of PC-12 cells with GFP-LC3 puncta formation were shown. Right: bar chart indicated the percentage of cells with GFP-LC3 puncta formation under the indicated treatments. (**d**) PC-12 cells, (**e**) wild type *Atg*7 and *Atg*7-deficient MEFs were treated with onjisaponin B (5 μM), *RP* (TEE) (62.5 μg/mL), *RP* (NF) (62.5 μg/mL) and *RP* (70–80% MF) (62.5 and 125 μg/mL) for 24 h. Cell lysates were then harvested and analyzed for LC3 I/II and β-actin, respectively. (**f**) Wild type *Atg*7 and *Atg*7-deficient MEFs transfected with GFP-LC3 were treated with onjisaponin B (5 μM), *RP* (TEE) (62.5 μg/mL), *RP* (NF) (62.5 μg/mL) and *RP* (70–80% MF) (62.5 and 125 μg/mL) for 24 h. Representative images of the cells with GFP-LC3 puncta formation were shown. Right: bar chart indicated the percentage of cells with GFP-LC3 under the indicated treatments. (**g**) Annexin V flow cytometry analysis in PC-12 cells after 24 h of *RP* (70–80% MF) treatments. Right: bar chart indicated the cell viability under these treatments. Data from the flow cytometry analysis is represented as means ± S.D. of three independent experiments. Bars, S.D. ***p < 0.001; **p < 0.01; *p < 0.05. Magnification, ×40; Scale bar: 15 μm. The full-length blots are presented in Supplementary Figure 5.

Previously, we have reported for the first time the autophagic and neuroprotective effect of *RP* (TEE) (500 μg/mL) and onjisaponin B (12.5 μM). In the current study, with the identification of the partially purified active fraction of *RP* by using CMC and UHPLC-(Q)TOF-MS, we therefore aim at comparing the autophagic and neuroprotective effect of the isolated *RP* fractions, including *RP* (TEE), *RP* (70–80% MF) and *RP* (NF) with onjisaponin B. Firstly, by using UHPLC-TOF-MS analysis and standard curve deduction (Fig. 6a,b), the concentration of onjisaponin B presents in the *RP* (70–80% MF) (62.5 μg/mL) and *RP* (NF) (62.5 μg/mL) was 4.45 μM and 1.62 μM, respectively. Therefore, 5 μM of onjisaponin B was used for comparison in all the biological assays. As shown in Fig. 6c,d, while both *RP* (70–80% MF) and *RP* (NF) increased the number of cells with GFP-LC3 puncta, 5 μM of onjisaponin B induced very weak autophagic effect in PC-12 cells. The result therefore supported our hypothesis that addition active compounds present in *RP* (70–80% MF) and *RP* (NF) may work as novel autophagic inducers, or work to enhance the autophagic effect of onjisaponin B.

To further study the molecular mechanisms of the isolated bioactive *RP* fractions, we investigated the autophagic effect of onjisaponin B (5 μM), *RP* (70–80% MF) (62.5 and 125 μg/mL), *RP* (NF) (62.5 μg/mL) and *RP* (TEE) (62.5 μg/mL) in both *Atg*7-wild type or -knockout mouse embryonic fibroblasts (MEFs) (Fig. 6e,f), which are resistant to autophagy induction^38^. *Atg*7 works as a putative regulator of autophagic function through mediating the autophagosomal biogenesis. Our results showed that both *RP* (70–80% MF) and *RP* (NF) induced autophagy in *Atg*7-wild type but not knockout MEFs. The result suggested that the identified bioactive fraction of *RP* induced autophagy through the autophagy related gene 7 (*Atg*7) dependent mechanisms. Furthermore, the cytotoxicity of the different isolated active fractions of *RP* on PC-12 cells was evaluated by flow cytometer. Without obvious cytotoxicity induced after treatments (Fig. 6g), our results suggested the high potential for applying the isolated autophagic fractions of *RP* to modulate neurodegenerative diseases, which are closely associated with the induction of autophagy^21^.

To further study the potential autophagic role or synergetic effect of onjisaponin B and other saponins in the active *RP* (70–80% MF), the active fraction was furthered separated into 6 sub-fractions (Sub-F1 to Sub-F6) (Supplementary Figure 2a), which were analyzed by UHPLC-TOF-MS for the percentage (as represented by the area of the peak) of different saponins. As shown in Supplementary Figure 2b and Supplementary Table 2, only 1.62%, 4.96% and 3.94% of onjisaponin B were presented in the sub-fraction 3, 4 and 5, respectively. On the other hand, sub-fraction 3 contained onjisaponin J (23.76%) and onjisaponin O (37.95%). In sub-fraction 4, 62.99% of chemical components were identified as polygalasaponin XXXII, onjisaponin J and onjisaponin R. Onjisaponin Fg, onjisaponin F and onjisaponin E contributed to 77.46% of total components in sub-fraction 5. In addition, Supplementary Figure 2c and Supplementary Table 3 showed the percentage of each saponin presented in each sub-fraction. For example, 94.16% of onjisaponin L were presented in sub-fraction 1. All the above results have provided useful information for us to evaluate the possible major components that were responsible for the bioactivity of the isolated active *RP* (70–80% MF).

To evaluate the potential autophagic role or synergetic effect of onjisaponin B and other saponins presented in the active *RP* (70–80% MF), the cytotoxicity (IC_50_ value) of sub-fractions 1 to 6 were first measured (Supplementary Figure 3a). All sub-fractions were determined to have an IC_50_ value >172 μg/mL, which is relatively non-toxic in nature. PC-12 cells transfected with GFP-LC3 were then treated with onjisaponin B, *RP* (70–80% MF) or the sub-fractions (1 to 6) respectively, for autophagy detection. As shown by western blot (Supplementary Figure 3b) and immunofluorescence microscopic analysis (Supplementary Figure 3c), all sub-fractions (1 to 6) which contained only small percentage of onjisaponin B, increased the expression of LC3 II and GFP-LC3 puncta formation to a similar extent as *RP* (70–80% MF). The results have further confirmed that other type of saponins contributed to the autophagic activity of *RP* (70–80% MF), and induced more potent autophagic activity when compared to onjisaponin B (5 μM).

### The active fractions of *RP* attenuates the protein level of mutant huntingtin

**Figure 7:**
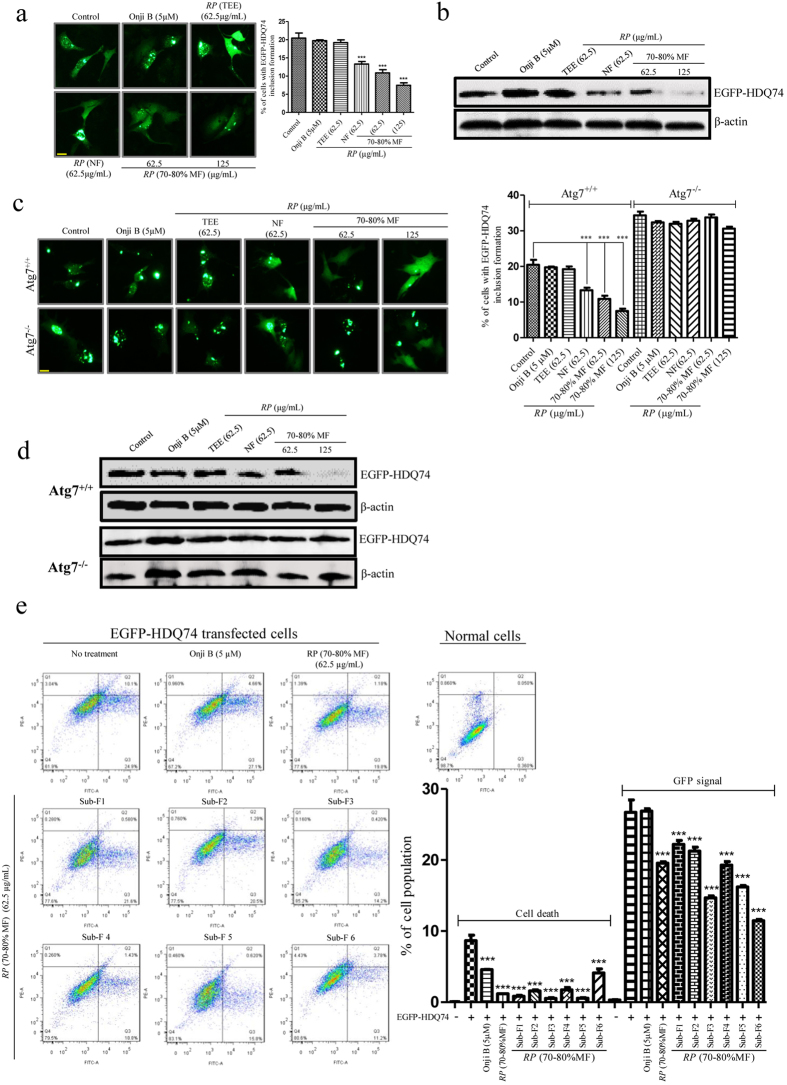
*RP* (70–80% MF) accelerated the clearance of mutant huntingtin. (**a**) PC-12 cells transfected with EGFP-HDQ 74 were treated with onjisaponin B (5 μM), *RP* (TEE) (62.5 μg/mL), *RP* (NF) (62.5 μg/mL) and *RP* (70–80% MF) (62.5 and 125 μg/mL) for 24 h before fluorescent microscopy analysis. Representative images showed the formation of EGFP-HDQ 74 inclusions. Right: bar chart indicated the percentage of cells with EGFP-HDQ 74 inclusions formation under the indicated treatments. (**b**) PC-12 cells transfected with EGFP-HDQ 74 were treated with onjisaponin B (5 μM), *RP* (TEE) (62.5 μg/mL), *RP* (NF) (62.5 μg/mL) and *RP* (70–80% MF) (62.5 and 125 μg/mL) for 24 h. Cell lysates were then harvested and analyzed for GFP and β-actin, respectively. (**c**) Wild type *Atg*7 and Atg7-deficient MEF cells transiently transfected with EGFP-HDQ 74 were treated with onjisaponin B (5 μM), *RP* (TEE) (62.5 μg/mL), *RP* (NF) (62.5 μg/mL) and *RP* (70–80% MF) (62.5 and 125 μg/mL) for 24 h before subjected to fluorescent microscopy analysis. Representative images of the cells with EGFP-HDQ 74 inclusions formation were shown. Right: bar chart indicated the percentage of cells with EGFP-HDQ 74 inclusions formation under the indicated treatments. (**d**) Wild type *Atg*7 and Atg7-deficient MEF cells transfected with EGFP-HDQ 74 were treated with onjisaponin B (5 μM), *RP* (TEE) (62.5 μg/mL), *RP* (NF) (62.5 μg/mL) and *RP* (70–80% MF) (62.5 and 125 μg/mL) for 24 h. Cell lysates were then harvested and analyzed for GFP and β-actin, respectively. (**e**) PC12 cells transfected with EGFP-HDQ 74 for 6 h were treated with onjisaponin B (5 μM), *RP* (70–80% MF) (62.5 μg/mL) or its sub-fractions 1–6 (62.5 μg/mL) for a further 24 h. Cells were then subjected to flow analysis after propidium iodide staining for cell death and GFP (FITC) signals detection. Statistically significant difference with respect to control or EGFP-HDQ 74 transfected cells were expressed as ***p < 0.001. Magnification, ×40; Scale bar: 15 μm. The full-length blots are presented in Supplementary Figure 6.

Neurode-generative diseases such as Huntington’s disease (HD) are caused by the accumulation of oligomeric or aggregate-prone toxic proteins in cells^39^,^40^. For example, HD is caused by the formation of long mutant huntingtin due to repeated CAG trinucleotide expansion. These long polyglutamine tract expansions are known to be associated with protein aggregate formation and cellular toxicity^41^,^42^. In our previous study, we reported that both *RP* (TEE) and onjisaponin B enhanced the degradation of mutant huntingtin. With a stronger autophagic effect induced by the isolated active fractions of *RP*, we therefore compared the neuroprotective effects of *RP* (70–80% MF), *RP* (NF) and *RP* (TEE) with onjisaponin B. To begin, we overexpressed mutant huntingtin plasmids with 74 CAG trinucleotide repeats (EGFP-HDQ 74) in PC-12 cells. In Fig. 7a,b, while 5 μM of onjisaponin B treatment showed no significant reduction in both protein and inclusion level of EGFP-HDQ 74, both *RP* (70–80% MF) and *RP* (NF) attenuated the level of EGFP-HDQ 74 as revealed by both fluorescent microscopy and western blot analysis.

To evaluate the protective autophagic effect of the fractions of *RP*, both *Atg*7-wild type and -knockout MEFs were transfected with EGFP-HDQ 74 plasmids for the inclusion formation of mutant huntingtin. Our results suggested that both *RP* (70–80% MF) and *RP* (NF) enhanced the degradation of EGFP-HDQ 74 inclusions and reduced its protein level in *Atg*7-wild type MEFs, but not *Atg*-deficient MEFs (Fig. 7c,d). The results further suggested that the neuroprotective effect of the isolated active *RP* fractions may depend on the induction of autophagy which requires the Atg7 gene.

It was confirmed that sub-fractions (1 to 6) which contained only small percentage of onjisaponin B, attenuated the inclusion (Supplementary Figure 3d) and protein level of huntingtin (EGFP-HDQ 74) (Supplementary Figure 3e) and A53T mutant α-synuclein (Supplementary Figure 3f) in cells. The results further supported our postulation that other than onjisaponin B, various type of saponins which shared a similar chemical structure and with high abundance in sub-fractions (1 to 6), may work together and contribute to the autophagic and neuroprotective effect of *RP* (70–80% MF).

The protective effect of *RP* (70–80% MF) and its 6 sub-fractions (1 to 6) on mutant huntingtin-induced cell death was further investigated^43^. As measured by flow analysis, the percentage of GFP-positive cells with the overexpression of mutant huntingtin was significantly lower in *RP* (70–80% MF) and its 6 sub-fractions (1 to 6) treatment groups when compared with onjisaponin B (5 μM) (Fig. 7e). In addition, the percentage of cell death was decreased after treatment of *RP* (70–80% MF) or sub-fractions (1 to 6) (Fig. 7e). The results suggested that the active *RP* (70–80% MF) may play potential therapeutic role in working as a neuroprotective agent, which reduced mutant proteins induced cell death in cells. Besides, the protective effect of sub-fractions (1 to 6) has further suggested the possible neuroprotective role of other saponins that were presented in *RP* (70–80% MF).

### The active fractions of *RP* accelerate the degradation of mutant α-synuclein and attenuate the oligomerization of α-synuclein in cells

**Figure 8:**
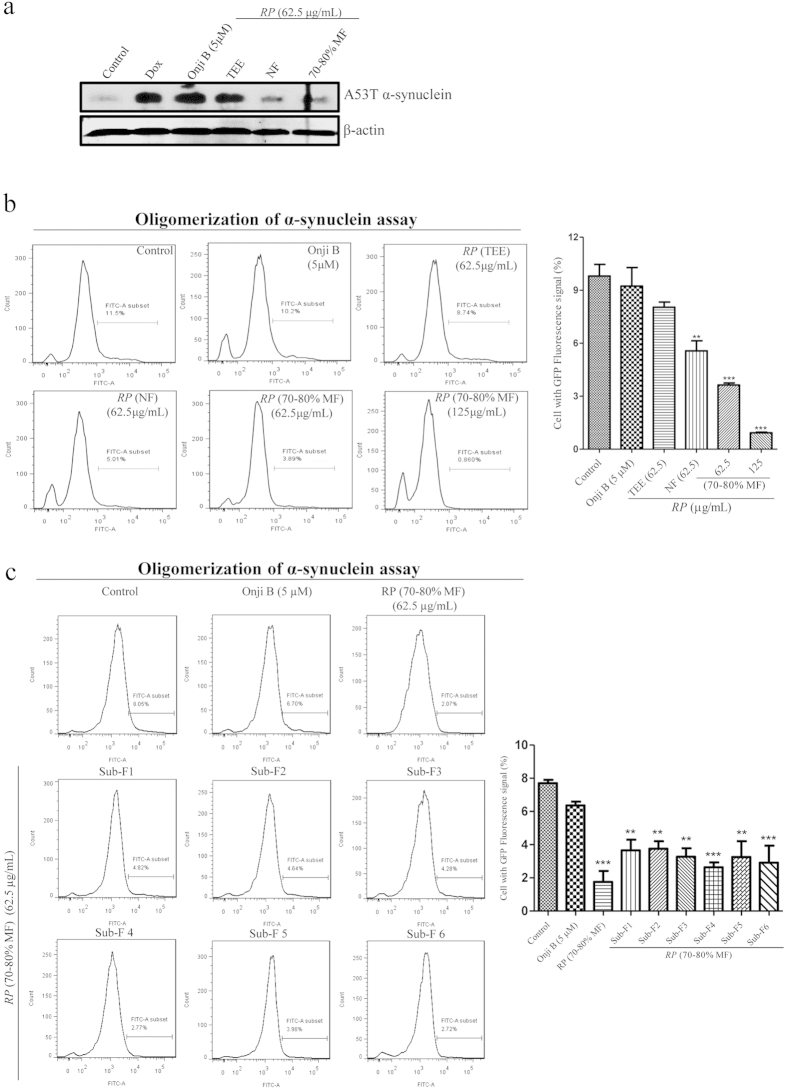
*RP* (70–80% MF) accelerated the clearance of α-synuclein. (**a**) PC-12 cells transiently transfected with α-synuclein were treated with onjisaponin B (5 μM), *RP* (TEE) (62.5 μg/mL), *RP* (NF) (62.5 μg/mL) and *RP* (70–80% MF) (62.5 μg/mL) for 24 h. Cell lysates were then harvested and analyzed for α-synuclein and β-actin, respectively. (**b**) HeLa cells transfected with both GNS and SGC plasmids were incubated with onjisaponin B (5 μM), *RP* (TEE) (62.5 μg/mL), *RP* (NF) (62.5 μg/mL) and *RP* (70–80% MF) (62.5 and 125 μg/mL), or (**c**) *RP* (70–80% MF) (62.5 μg/mL) and *RP* (70–80% MF) sub-fractions (1–6) (62.5 μg/mL) for 24 h. The percentage of cells with α-synuclein oligomerization was assessed by flow cytometry. Bar chart indicated the percentage of cells with green fluorescent protein (GFP)-positive signal under these treatments. Data from the flow cytometry analysis is represented as means ± S.D. of three independent experiments. Bars, S.D. ***p < 0.001; **p < 0.01. The full-length blots are presented in Supplementary Figure 7.

Aggregated α-synuclein may contribute to Parkinson’s disease (PD) through its oligomeric conformation, which leads to the disruption of cellular homeostasis and neuronal death^44^. A53T mutant α-synuclein is also known to be associated with the pathogenesis of PD^42^,^45^. Therefore, we examined if the isolated autophagic fractions of *RP* accelerate the degradation of A53T mutant α-synuclein (autophagy substrate), by using a doxycycline (Dox) -inducible cellular model. Firstly, the expression of A53T mutant α-synuclein was induced by the addition of Dox^21^. We then evaluated if *RP* (70–80% MF) and *RP* (NF) were able to enhance the degradation of A53T mutant α-synuclein in PC-12 cells. In Fig. 8a, while both *RP* (70–80% MF) and *RP* (NF) accelerated the degradation of A53T mutant α-synuclein (tagged with myc), 5 μM of onjisaponin B did not attenuate the protein level of α-synuclein after the induction of Dox. Collectively, the results further confirmed the additional active components presented in *RP* (70–80% MF) or *RP* (NF), may exhibit more potent biological effect than onjisaponin B. Through accelerating the degradation of mutant proteins in cellular assay, the isolated active autophagic fraction of *RP* containing 17 major chemical components, may act as a potential neuroprotective agent in the future.

In addition to mutation that may lead to misfold of α-synuclein, oligomerization of α-synuclein may also contribute to the pathogenesis of Parkinson’s disease (PD)^44^,^46^,^47^. To this end, α-synuclein proteins fused with non-fluorescent GFP-N terminal (GNS) or -C terminal (SGC) fragments were used to monitor the oligomerization of α-synuclein *in vitro*. As oligomerization of α-synuclein will reconstitute the non-fluorescent incomplete GFP fragments into complete GFP fluorophore, and give GFP fluorescent signal as monitored by flow cytometer, both *RP* (70–80% MF) and *RP* (NF) inhibited the oligomerization of α-synuclein in cells overexpressed with GNS and SGC plasmids (Fig. 8b). Through inhibiting the oligomerization of α-synuclein in cells, the decrease in the GFP fluorescent signal indicated that both *RP* (70–80% MF) and *RP* (NF) may have protective effect in PD. Consistently, more potent effect on the reduction of α-synuclein oligomerization was observed in *RP* (70–80% MF) and *RP* (NF) when compared with equal amount of onjisaponin B alone. In addition, sub-fractions (1 to 6) also demonstrated potent effect on the reduction of α-synuclein oligomerization when compared with onjisaponin B (Fig. 8c). All these results therefore supported our postulation that additional active saponins are responsible for the protective effect of *RP* (70–80% MF).

## Discussion

Recent literatures have reported the important role of CHM in the prevention and treatment of the neurodegenerative diseases such as Parkinson’s Disease, Alzheimer’s Disease and Huntington’s Disease^48^,^49^. Risk factors or symptoms such as aging, depression, epilepsy, forgetful and slowness in movement are highly correlated to neurodegeneration^50^,^51^,^52^,^53^. With its protective function in reinforcing heart, tranquilization, relieving convulsion and spasm, *RP* is commonly used in CHM prescriptions for the treatment of neurodegenerative disorders^24^,^25^.

From our previous study, it was shown that both *RP* (TEE) (500 μg/mL) and onjisaponin B (25 μM) induced autophagy and accelerated the degradation of A53T mutant α-synuclein and mutant huntingtin (74 CAG repeats) in cellular models. As many of the triterpenoid saponins present in the extract of *RP* share a similar chemical structure as onjisaponin B, we therefore investigated whether additional compounds from *RP* are also able to exhibit neuroprotective effect through autophagy. To this end, we first performed the CMC analysis to identify the chemical components which have the binding affinity to the membrane of PC-12. After analyzed by high sensitive UHPLC-(Q)TOF-MS, we finally identified 17 major chemical components present in the CMC-isolated fraction of *RP*.

To confirm the CMC results, we then performed chemical extraction and column chromatography to obtain the cellular membrane binding fraction of *RP*. Our preliminary data showed that the membrane binding fraction (C5) isolated by CMC analysis was able to induce autophagy at a higher extent than onjisaponin B (5 μM) alone. Therefore, we suggested that additional active compounds may present in the C5 fraction of *RP*, or some compounds in the C5 fraction may work to enhance the autophagic effect of onjisaponin B. In fact, because of the complexity of the chemical constituents and molecular mechanism of CHM, it is always prescribed as decoction or soup instead of single component for the treatment of diseases. In this study, our results have therefore suggested the possible pharmacological mechanisms and autophagic functions of purified extract of *RP*, which is a top grade medicine in Chinese history. In addition, through the purification of *RP* (TEE) by the methanol or the n-butanol and ethylacetate solvent systems, our findings may help to further improve the efficacy of current composition and formulation of medical products of *RP*.

Our result further demonstrated that *RP* (70–80% MF), which is composed of 17 major chemical components, induced autophagy in a higher extent than onjisaponin B alone. Other fractions of *RP* eluted by using different percentage of methanol (10% to 60% or 90 to 100%) have only very weak or no autophagic effect. This result is consistent with the UHPLC-TOF-MS analysis which showed that only very few chemical components are presented in these fractions. The identities of the 17 chemical components in *RP* (70–80% MF) were further confirmed by using UHPLC-TOF-MS and UHPLC-Q-TOF-MS to obtain their accurate MS, molecular formula and the main characteristic fragment ions. However, it is reported that onjisaponin V and onjisaponin Vg share the same MS as they have the same aglycone presenegenin and sugar residues^19^. Therefore, peak 5 can be identified as onjisaponin Vg or onjisaponin V with the molecular formula of C_82_H_122_O_41_, although the positions of R1 and R2 in onjisaponin Vg and V are different. Therefore, further purification on peak 5, and the study on chemical shift of H-4 and H-3 in fucose between onjisaponin V and onjisaponin Vg by using nuclear magnetic resonance (NMR) may be required in the future^19^.

With the advantage of rapidity, accuracy and simplicity, we have reported for the first time the novel application of using CMC to identify new autophagy inducers with neuroprotective effects from CHM. When evaluating the potential autophagic and neuroprotective role of other type of saponins that were presented in *RP* (70–80% MF), we have further separated *RP* (70–80% MF) into 6 sub-fractions for investigation. However, as the saponins of *RP* are mainly oleanolic acid type pentacyclic triterpenoid saponins with very close polarity, this make the isolation and purification procedures of different saponins very difficult. Furthermore, the sugar chains in these saponins usually contain the cinnamoyl substituent, which the cis-trans isomers of cinnamoyl are easy to interconvert. All these factors may affect the stability of saponins in *RP* and therefore, trace amount of onjisaponin B is still remained in the *RP* sub-fractions 3, 4 and 5, although the concentration and percentage of onjisaponin B in those sub-fractions are much lower than in *RP* (70–80% MF).

Triterpenoid saponins including polygalasaponin and onjisaponin are the characteristic components in *Polygala*. Here, the isolated autophagic *RP* (70–80% MF) fraction, which contains 17 kinds of saponins, is proved to be effective in enhancing the removal of mutant proteins in cells. Although 17 major types of saponins in *RP* (70–80% MF) were identified by using CMC and UHPLC-(Q)TOF-MS, some other type of very low abundance saponins which were not identified by CMC or UHPLC-(Q)TOF-MS, may also induce autophagy and accelerate the degradation of mutant proteins in cells due to their similar chemical structures. In theory of CHM, the characteristic components of medicinal herb are defined as the components exist only in this herb, which may contribute to the therapeutic effects of the plants. For example, flavonoids and terpene lactones are the characteristic components responsible for the medicinal effects of *Ginkgo biloba*/EGb 761® (EGb 761), which is used to treat cardiovascular diseases, loss of memory and cognitive disorders associated with age-related dementia^54^. Valerenic acids are the characteristic components of valerian, which has sedative effects and improves sleep quality^55^. Therefore, valerenic acids are used as chemical markers to evaluate the quality of valerian preparations. For the quality control of *RP*, we monitored the content of the 17 kinds of saponins by measuring the concentration of the total saponins present in *RP* (70–80% MF), with tenuifolin as the standard control according to the “Chinese Pharmacopoeia 2010”. Therefore, with the fact that the partially purified autophagic *RP* (70–80% MF) exhibits potent autophagic and neuroprotective effect, therefore, our current study not only provides novel insights into the pharmacological actions of *RP*, which improve the current way of prescribing *RP* decoction, but also introduces a new standard for the quality control of the *RP* decoction in the commercial market.

## Methods

### Reagents, chemicals, antibodies and plasmids

*RP* was purchased from Kangmei Pharmaceutical Co., Ltd. (Guang Dong, China), onjisaponin B and tenuifolin were obtained from MUST Bio-technology Company Ltd. (Chengdu, China). The ODS packing materials were purchased from Grace Davison Discovery Sciences (formerly Alltech Associates, Deerfield, IL). Unless otherwise specified, all reagents or chemicals were obtained from Sigma-Aldrich (MO, USA). EGFP-LC3 plasmid was kindly provided by Tamotsu Yoshimori (Osaka University, Osaka, Japan). EGFP-HDQ 74 plasmid was gift from David C. Rubinsztein (University of Cambridge, Cambridge, UK). Myc-tag and LC3-B antibodies were obtained from Cell Signaling Technologies Inc. (MA, USA). Antibodies against GFP and β-actin were obtained from Santa Cruz Biotechnology (CA, USA) and Sigma-Aldrich (MO, USA), respectively.

### Instruments and chromatographic conditions

UHPLC (Agilent Technologies 1290 Series) equipped with the time of flight MS (Agilent Technologies 6230) with a jet stream ion source was operated in negative ion mode during the UHPLC analysis.

The samples were analyzed by using the Agilent Zorbax Eclipse Plus C-18 column with a particle size of 1.8 μm (flow rate: 0.35 mL/min). The parameters of the gradient elution program were applied as follows: mobile phase A (0.1% formic acid in water) and mobile phase B (0.1% formic acid in ACN): 0–2 min, 2% B; 2–5 min, 2–10% B; 5–15 min, 10–50% B; 15–18 min, 50–80% B; 18–20 min, 80–100% B; 20–22 min, 100% B; 22.1–25 min, 2% B. For UHPLC-TOF-MS analysis, the data was acquired in the scan mode (m/z 100 to 3200 Da with 2.0 spectra/s). Data were analyzed by using Agilent MassHunter Workstation software B.01.03.

UHPLC-Q-TOF-MS was performed on the Agilent series 1290 UHPLC instrument coupled with a 6550 Q-TOF mass spectrometer in negative ion mode, by using the same mobile phase and flow rate as in the UHPLC-TOF-MS analysis. The acquisition parameters were set as follows: drying gas (N2) flow rate, 11.0 l/min; temperature, 250 °C; nebulizing gas, 40 psig; capillary, 3500 V; fragmentor, 175 V; skimmer, 60 V; OCT RF V,750 V. The collision energy (CE) was set 65 V and the mass range recorded m/z 100–3200 with a resolution 15,000. Data were analyzed by using Agilent MassHunter Workstation software B.01.03.

### Preparation of total ethanol extract (TEE) of *Radix Polygalae* (*RP*)

30 g of *RP* powder was first immersed in 300 mL of 75% ethanol for 1 h, and then refluxed twice with 75% ethanol for 1 h respectively. The solvent was removed by rotary evaporation at 60 °C under vacuum condition. The residue was then re-dissolved in DMSO until further use.

### Cell membrane chromatography (CMC)

TEE of *RP* at a final concentration of 125 to 750 μg/mL were incubated with PC-12 cells for 1 to 6 h. After incubation, the supernatant was transferred to a 15 mL tube and the remaining cells were washed with PBS for 5 times. The final PBS washing solution was collected as the control to confirm all compounds that without binding affinity to cell membrane were washed away completely. The remaining cells were then lysed by using 3 mL of citric acid buffer (pH 4.0) at 37 °C^30^. To facilitate complete cellular disruption, the cells suspension was subjected to ultrasound sonication for further disruption. The denatured cells were centrifuged with 2500 rpm for 5 min and the supernatant was collected and dried by nitrogen. The residue was then re-dissolved by methanol and filtered through a 0.22 μm microporous membrane before analysis^30^.

### Isolation and purification of the active methanol fraction (MF) of *RP* by using the methanol gradient system

2 isolation methods were applied to purify and isolate the *RP* fraction which has high binding affinity to the PC-12 cellular membrane as revealed by CMC analysis. Firstly, ODS column was used to purify the TEE of *RP* by eluting its different chemical components gradually with water and increasing percentage of methanol (10%–100%). After elution, a total of 11 fractions were collected and dried for UHPLC-TOF-MS analysis. As a comparison, the *RP* (TEE) was concentrated, dried and re-dissolved in water, and then partitioned with ethylethanoate (1:1 vol/vol) for 3 times. The ethylethanoate fraction was evaporated and the remaining water layer was partitioned with n-Butanol (1:1 vol/vol) for 3 times to provide the n-butanol and water soluble fraction. Finally, water fraction (WF), n-Butanol fractions (NF) and ethylethanoate fraction (EF) of *RP* were collected and dried completely for further UHPLC-TOF-MS and biological activity analysis.

### Measurement of the amount of total saponins in the methanol fraction (MF) of *RP*

To determine the total amount of saponins present in the 70–80% MF of *RP*, tenuifolin was used as a standard control according to the standard protocol described in “Chinese Pharmacopoeia 2010”^37^,^56^. In brief, 70–80% MF of *RP* was dried to powder, which was re-dissolved in 10% NaOH solution and refluxed for 2 h. The solution was then cooled down and the pH value was calibrated to pH 4–5. Saturated n-butanol was used to extract the solution for 3 times and then the n-butanol fraction was dried by the rotary vacuum evaporator. The residue was re-dissolved in methanol and adjusted to a final volume of 50 mL. The tenuifolin standard solution and the methanol dissolved sample were filtered using 0.22 μm microporous membrane and analyzed by HPLC.

### Cell Culture

Unless otherwise specified, all cell lines were obtained from the American Type Culture Collection (ATCC) (Rockville, MD, USA). Both *Atg*7 -wild type and -knockout mouse embryonic fibroblasts (MEFs) were kindly provided by Masaaki Komatsu (Juntendo University, Tokyo, Japan). All cells were maintained in DMEM supplemented with fetal bovine serum (10%), penicillin (50 U/mL) and streptomycin (50 μg/mL) (Invitrogen, Scotland, UK) at 37 °C with CO_2_ (5%), except PC-12 cells which were maintained in horse serum (10%). Dox- inducible PC-12 cell line was cultured with DMEM supplemented with horse serum (10%), Tet system approved fetal bovine serum (5%) (Clontech, CA, USA) in the presence of G418 (200 μg/mL).

### Cytotoxicity Assays

MTT (3-[4,5-dimethylthiazol-2-yl]-2,5 diphenyl tetrazolium bromide) assay were performed to measure cell viability of cells as described in previous studies^57^. Cell viability was also measured by annexin V staining kit (BD Biosciences, San Jose, CA, USA). Cells after treatments were stained and analyzed by FACSCalibur flow cytometer (BD Biosciences). Data analysis and acquisition were performed with CellQuest (BD Biosciences).

### Quantification of GFP-LC3 Puncta formation

Cells treated with different *RP* fractions were first fixed with paraformaldehyde (4%), and then subjected to fluorescence microscopic analysis (Nikon ECLIPSE 80i microscope) with representative images captured by CCD digital camera Spot RT3™ (Diagnostic Instruments, Melville, NY). The number of GFP-positive cells with fluorescence LC3 puncta was examined and quantified as described in previous study^8^.

### Western Blot

Cells harvested with RIPA buffer (Cell Signaling Technologies Inc, MA, USA) were subjected to SDS/PAGE analysis. The proteins from SDS-PAGE were electrotransferred to protein membrane. Appropriate primary antibodies were then incubated with the membrane overnight with constant shaking. After incubation with secondary antibodies conjugated with HRP, protein bands were visualized by using the ECL Detection Reagents (Invitrogen, Paisley, Scotland, UK)^58^.

### Removal of mutant proteins

Cells were transfected with the EGFP-HDQ 74 plasmid for 24 h by using Lipofectamine® LTX and Plus™ reagent (Invitrogen, NY, USA). The EGFP-HDQ 74 transfected cells were then incubated with different fractions of *RP* for a further 24 h. The protein level of mutant huntingtin was then evaluated by western blotting using GFP antibodies. PC-12 cells transfected with EGFP-HDQ 74 plasmid were subjected to fluorescent huntingtin inclusion formation analysis after 24 h of *RP* treatments. Percentages of cells with fluorescent inclusion formation were counted^21^.

Dox-inducible PC-12 cells were first transfected with A53T mutant α-synuclein plasmids for 24 h. Transfected cells were incubated with 1 μg/mL of Dox (24 h) for the induced expression of A53T α-synuclein^21^. The overexpression of α-synuclein was then switched off by removing dox-containing medium from PC-12 cells. Cells were then treated with different fractions of *RP* for 24 h. The protein level of mutant α-synuclein was measured by western blot using myc-tag antibody. Both the dox -inducible PC-12 cell line and A53T mutant α-synuclein plasmids were provided by C.B. KO (The Hong Kong Polytechnic University, Hong Kong, China).

### Bimolecular Fluorescence Complementation (BiFC) Assay

Both GNS and SGC plasmids were gifts from Pamela J. McLean (Department of Neuroscience, Mayo Clinic Florida, Jacksonville, FL, USA). HeLa cells transfected with both GNS and SGC plasmids were incubated at 37 °C for 4 h^59^. The transfected cells were then treated with different fractions of *RP* for 24 h in a humidified incubator at 30 °C. Fluorescent signals were then detected by flow analysis (BD FACSAria III, San Jose, CA, USA).

### Statistical Analysis

All data were expressed as means ± S.D. as shown. The difference was considered statistically significant if the *p*-value < 0.05. Either one-way ANOVA or student’s *t*-test was used for comparison between different treatment groups.

## Additional Information

**How to cite this article**: Wu, A.-G. *et al*. Identification of novel autophagic *Radix Polygalae* fraction by cell membrane chromatography and UHPLC-(Q)TOF-MS for degradation of neurodegenerative disease proteins. *Sci. Rep*. **5**, 17199; doi: 10.1038/srep17199 (2015).

## Supplementary Material

Supplementary Information

## Figures and Tables

**Table 1:** The chemical name, formula, molecular weight and accurate MS of the 17 main compounds present in the *RP* fraction (C5) identified by the CMC and UHPLC-(Q)TOF-MS analysis.

Peak No	Chemical Name	Formula	Accurate MS
1	Onjisaponin B	C_75_H_112_O_35_	1572.698
2	Polygalasaponin XXXII	C_79_H_118_O_38_	1674.730120
3	Onjisaponin L	C_86_H_128_O_43_	1848.782945
4	Onjisaponin J	C_85_H_126_O_42_	1818.772380
5	Onjisaponin Vg/V	C_82_H_122_O_41_	1762.746165
6	Onjisaponin Fg	C_81_H_120_O_40_	1732.735600
7	Onjisaponin Ng	C_80_H_118_O_38_	1686.730120
8	Onjisaponin O	C_77_H_116_O_37_	1632.719555
9	Onjisaponin Gg	C_76_H_112_O_36_	1600.693340
10	Onjisaponin Y	C_69_H_102_O_30_	1410.645600
11	Onjisaponin H	C_74_H_110_O_34_	1542.687860
12	Senegasaponin A	C_74_H_110_O_35_	1558.682775
13	Onjisaponin R	C_76_H_114_O_37_	1618.703905
14	Onjisaponin F	C_75_H_112_O_36_	1588.693340
15	Senegasaponin B	C_69_H_102_O_31_	1426.641
16	Onjisaponin E	C_71_H_106_O_33_	1486.661645
17	Onjisaponin A	C_80_H_120_O_39_	1704.740685
