# Supplementary material for: Identification of novel autophagic Radix Polygalae fraction by cell membrane chromatography and UHPLC-(Q)TOF-MS for degradation of neurodegenerative disease proteins
Source: Sci Rep. 2015 Nov 24;5:17199. doi: 10.1038/srep17199 (PMC4657008; doi:10.1038/srep17199)
Supplement: Supplementary Information [file srep17199-s1.pdf]

Subject Area: Drug discovery

*Corresponding authors:** Dr. Betty Yuen Kwan Law and Prof. Liang Liu
Address correspondence: State Key Laboratory of Quality Research in Chinese Medicine, Macau University of Science and Technology, Avenida Wai Long, Taipa, Macau, China
Tel.: +853-8897 2238 (L.L.); +853-8897 2407 (B.Y.-K.L.); Fax: +853-2882 3312 (L.L.); +853-2882 7222 (B.Y.-K.L.)

E-mail address: yklaw@must.edu.mo (B.Y.-K.L.); lliu@must.edu.mo (L. Liu)

Identification of novel autophagic *Radix Polygalae* fraction by cell membrane chromatography and UHPLC-(Q)TOF-MS for degradation of neurodegenerative disease proteins

An-Guo Wu, Vincent Kam-Wai Wong, Wu Zeng, Liang Liu * and Betty Yuen-Kwan Law *

State Key Laboratory of Quality Research in Chinese Medicine, Macau University of Science and Technology, Macau, China

Supplementary Figure 1

MS/MS spectrum of 17 saponins

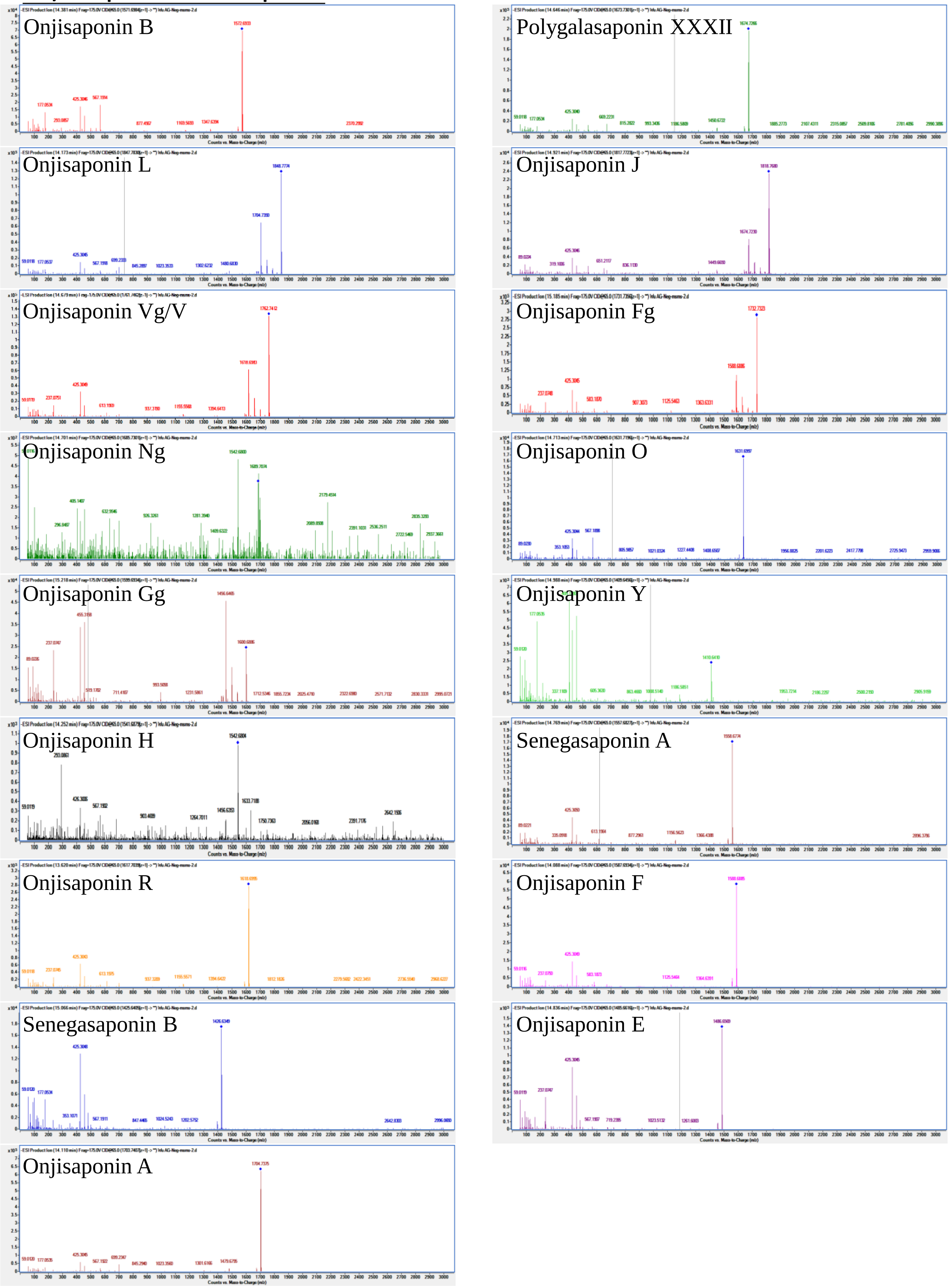

Supplementary Figure 2

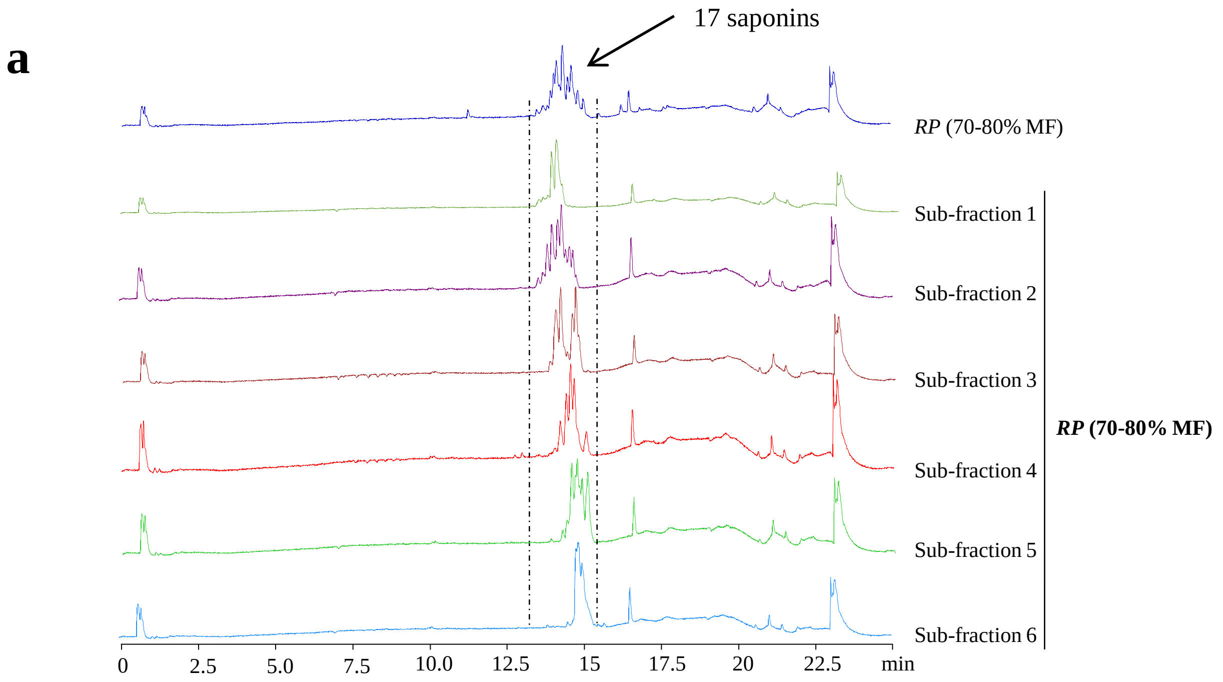

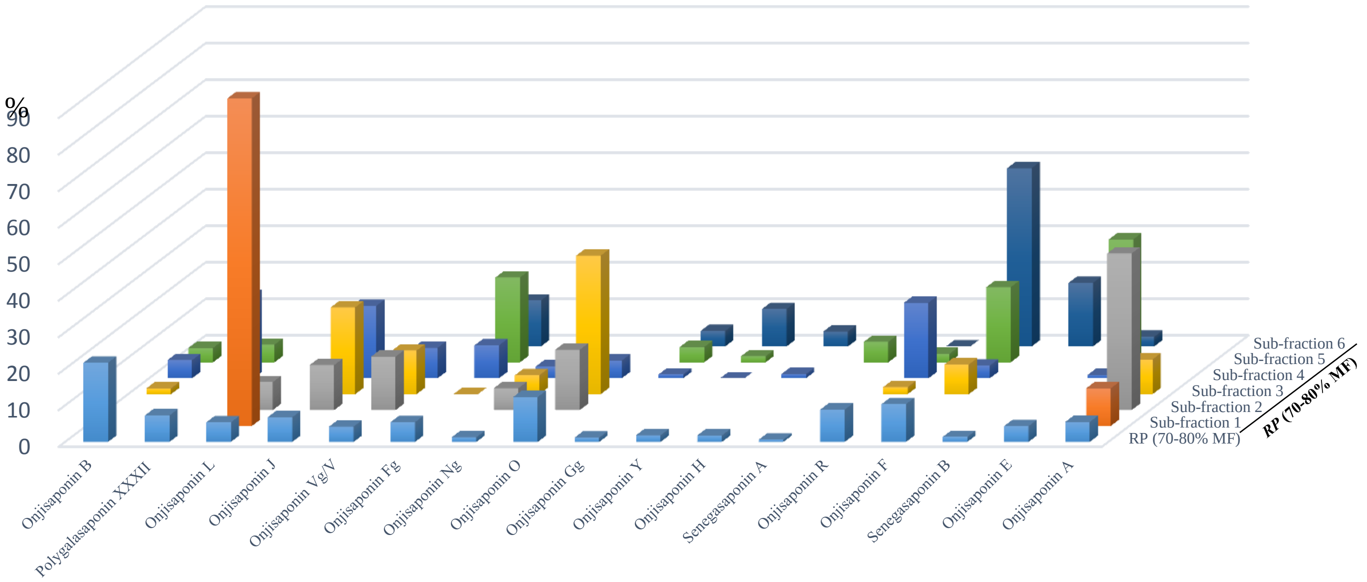

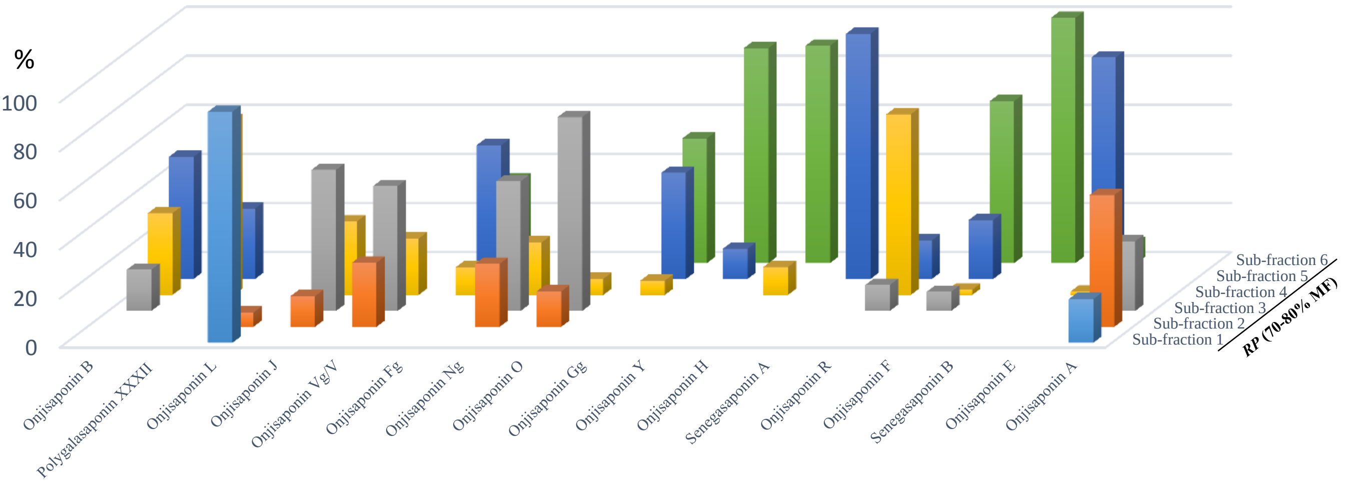

Supplementary Figure 2: The percentage of different type of saponins in *RP* (70-80% MF). (a) The TIC of *RP* (70-80% MF), and *RP* (70-80% MF) sub-fractions (1-6) using open column chromatography and preparative HPLC. (b) Each bar represented the percentage of different type of saponins presented in the isolated fractions. Numerical data (%) were shown in Supplementary Table 2. As shown in Supplementary Table 2, 21.68 % of onjisaponin B were presented in *RP* (70-80% MF). In sub-fraction 1, 89.68% of saponin was onjisaponin L. Onjisaponin J (12.33%), onjisaponin Vg/V (14.63%), onjisaponin O (16.52 %) and onjisaponin A (42.87%) contributed to 86.34% of total saponins in sub-fraction 2. In fraction 3, 61.7 % of saponins were onjisaponin J and onjisaponin O. Polygalasaponin XXXII (22.5%), onjisaponin J (19.8 %) and onjisaponin R (20.69 %) contributed to 62.99% of total saponins in sub-fraction 4. In sub-fraction 5, 77.46 % of total saponins were onjisaponin Fg, onjisaponin F and onjisaponin E. In sub-fraction 6, 78.77% of total saponins were onjisaponin Fg, onjisaponin F and Senegasaponin B. (c) The distribution of different saponins (%) in all sub-fractions (1-6). Numerical data (%) were shown in Supplementary Table 3. Onjisaponin L (94.16%) was mainly presented in sub-fraction 1. Onjisaponin A (53.75%) was mainly presented in sub-fraction 2. Onjisaponin J, onjisaponin Vg/V, onjisaponin Ng and onjisaponin O was mainly presented in sub-fraction 3. PolygalasaponinXXXII and onjisaponin R was mainly presented in sub-fraction 4. Onjisaponin Fg and onjisaponin O was mainly presented in sub-fraction 5. Onjisaponin Gg, onjisaponin Y, onjisponin H and onjisaponin F was mainly presented in sub-fraction 6.

Supplementary Figure 3

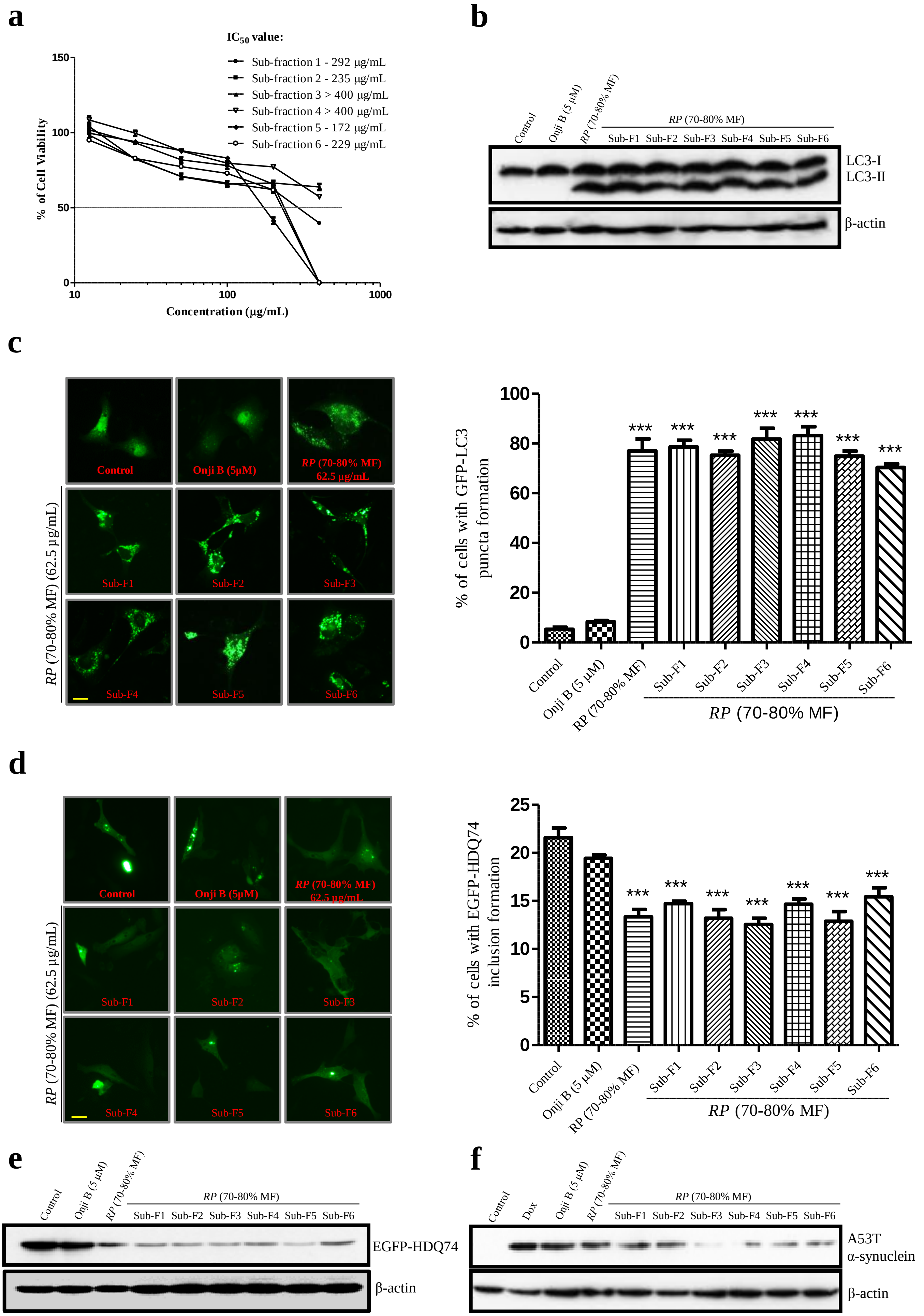

Supplementary Figure 3: The autophagic activity of *RP* (70-80% MF) and its sub-fractions (1-6) in PC-12 cells. (a) Cytotoxicity measurement (IC_{50} value) of the 6 isolated sub-fractions after 48 h of treatment. (b) PC-12 cells were treated with onjisaponin B (5 μM), *RP* (70-80% MF) (62.5 μg/mL) or its sub-fractions 1-6 (62.5 μg/mL) for 24 h. Cell lysates were then harvested and analyzed for expression of LC3 and β-actin. (c) PC-12 cells transiently transfected with GFP-LC3 were treated with onjisaponin B (5 μM), *RP* (70-80% MF) (62.5 μg/mL) or its sub-fractions 1-6 (62.5 μg/mL) for 24 h. Representative images and bar chart showed the formation and quantitation of GFP-LC3 puncta after treatments. (d) PC-12 cells transfected with EGFP-HDQ 74 were treated with onjisaponin B (5 μM), *RP* (70-80% MF) (62.5 μg/mL) or its sub-fractions 1-6 (62.5 μg/mL) for 24 h. Representative images and bar chart showed the formation and quantitation of EGFP-HDQ 74 inclusions after treatments. (e) PC-12 cells transfected with EGFP-HDQ 74 were treated with onjisaponin B (5 μM), *RP* (70-80% MF) (62.5 μg/mL) or its sub-fractions 1-6 (62.5 μg/mL) for 24 h. Cell lysates were then harvested and analyzed for expression of EGFP-HDQ74 and β-actin. (f) Dox-inducible PC-12 cells transfected with α-synuclein were treated with onjisaponin B (5μM), *RP* (70-80% MF) (62.5 μg/mL) or its sub-fractions 1-6 (62.5 μg/mL) for 24 h. Cell lysates were then harvested and analyzed for α-synuclein and β-actin, respectively. The full-length blots are presented in Supplementary Figure 8.

Supplementary Figure 4

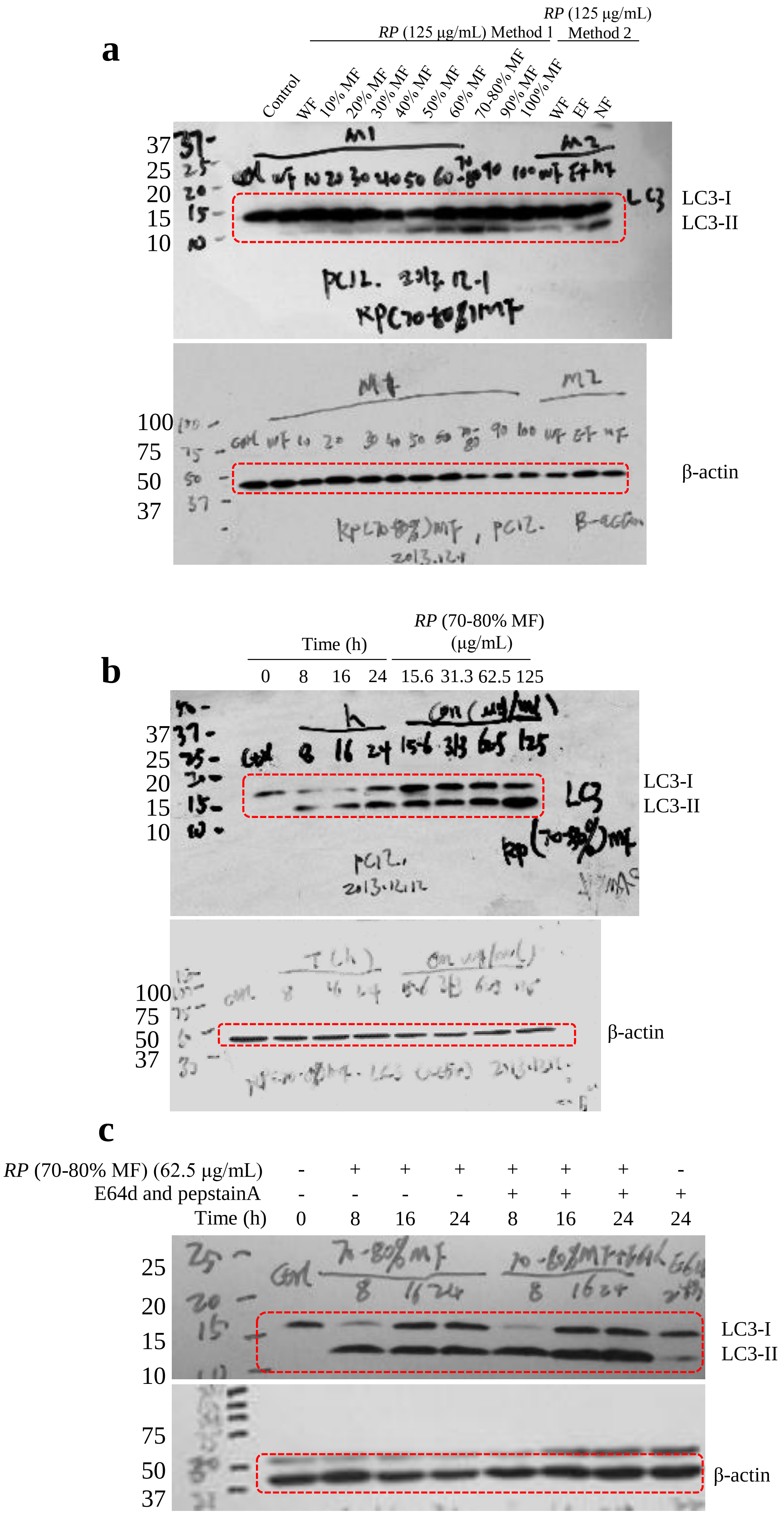

Supplementary Figure 5

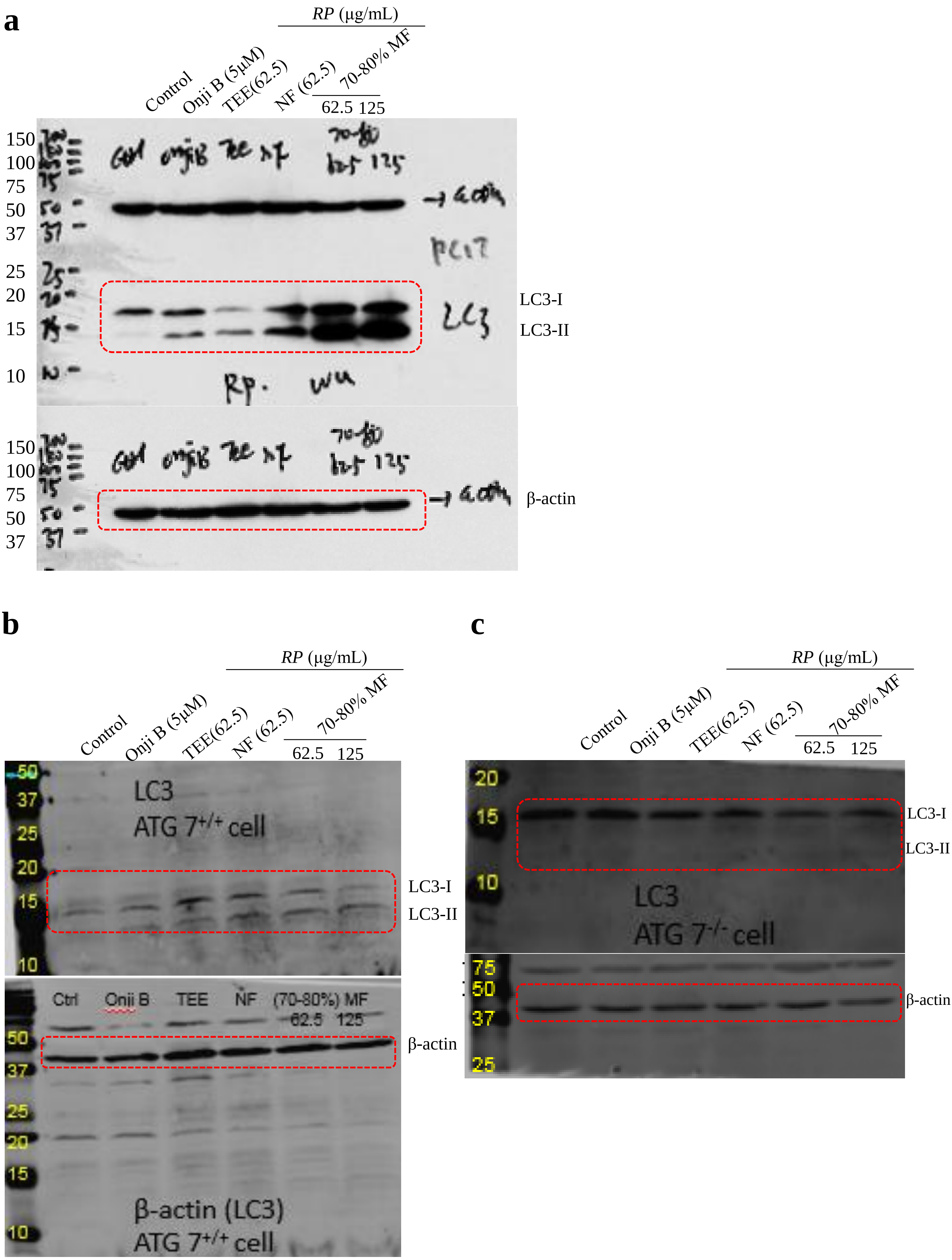

Supplementary Figure 6

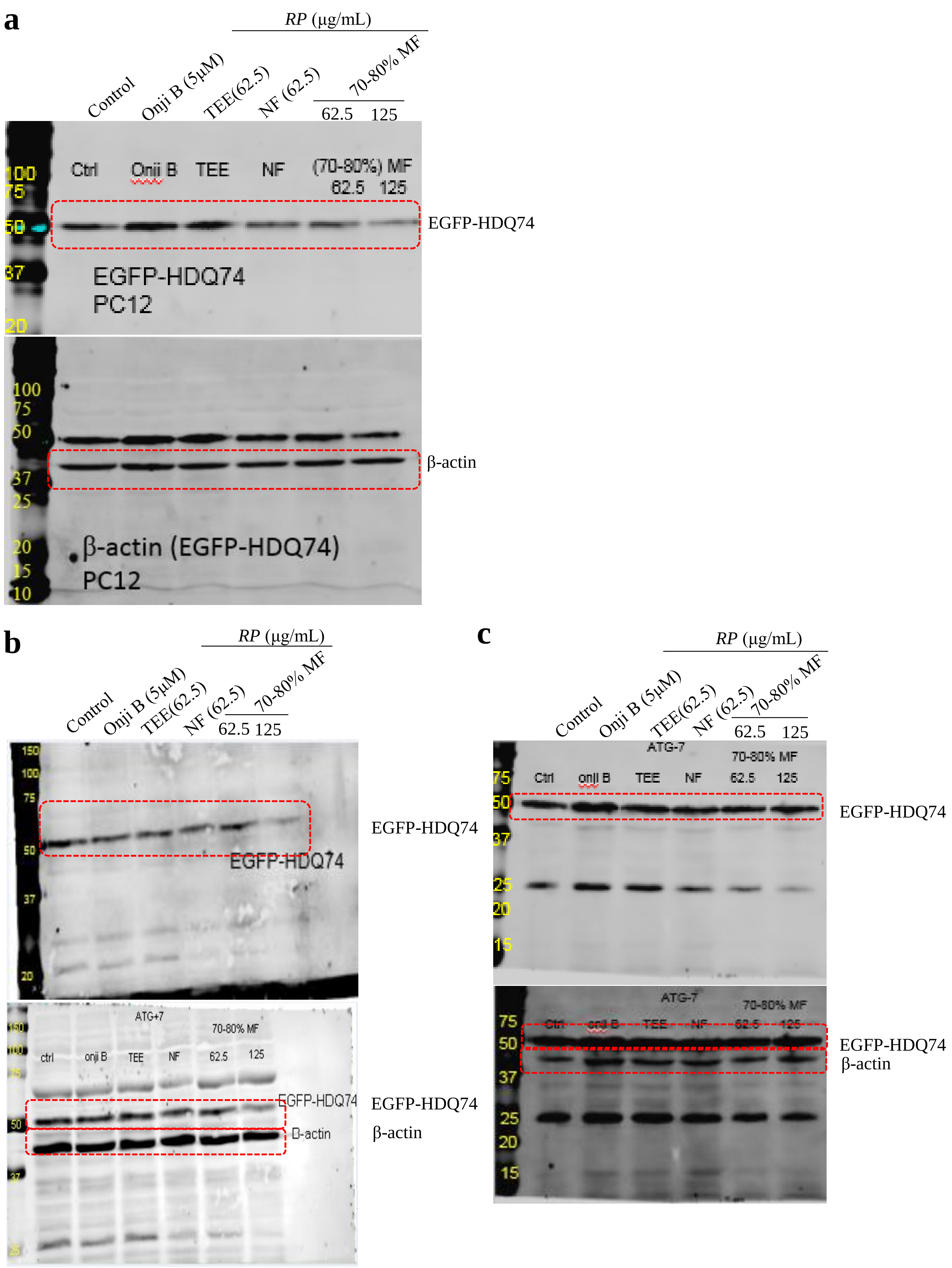

Supplementary Figure 7

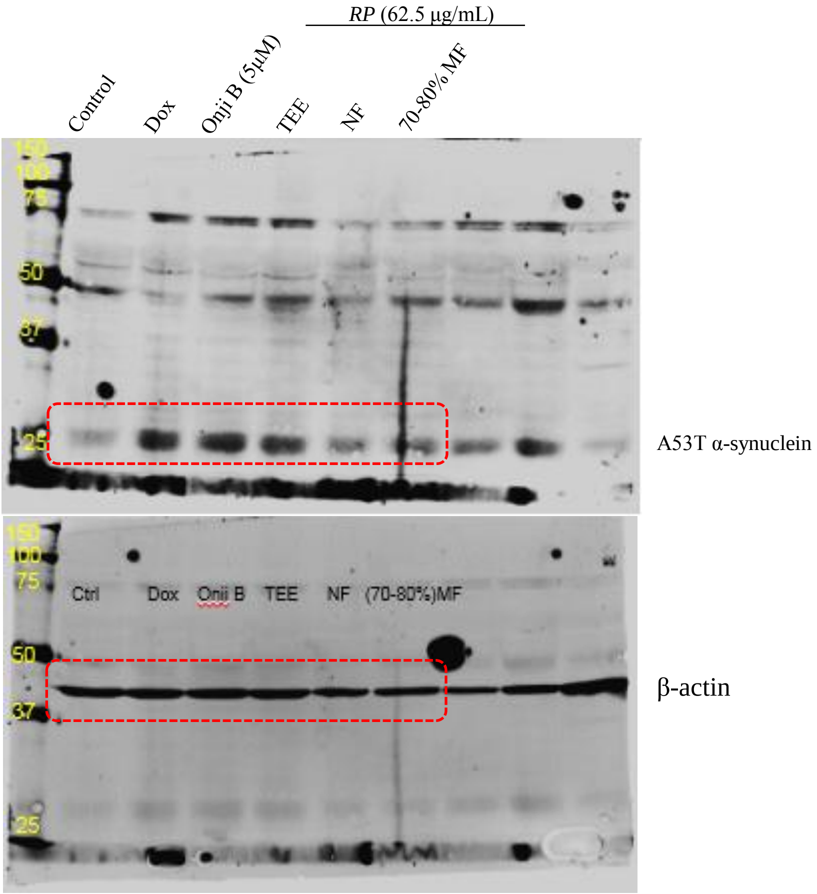

Supplementary Figure 8

a

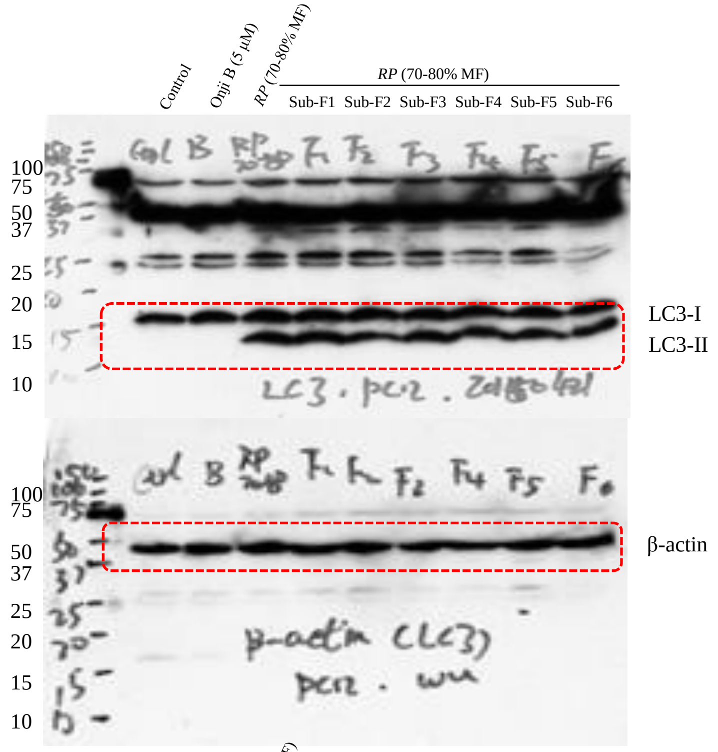

b

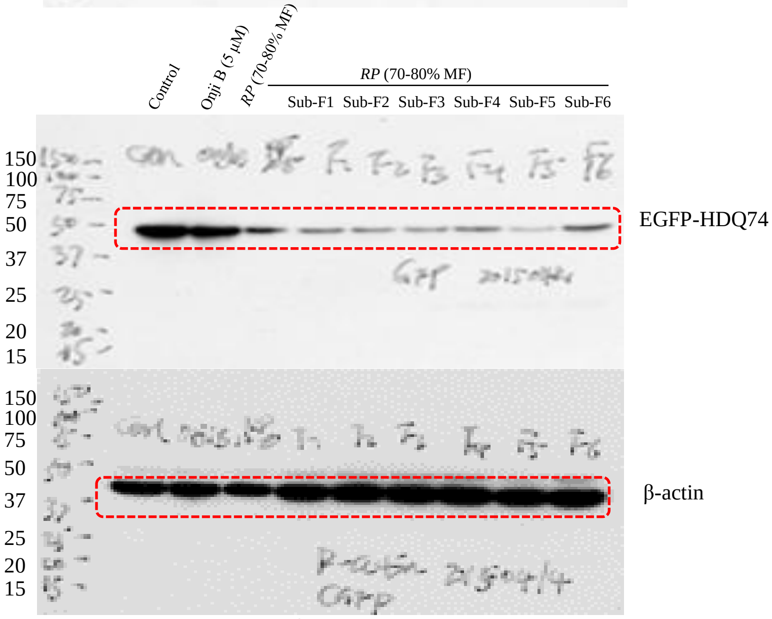

c

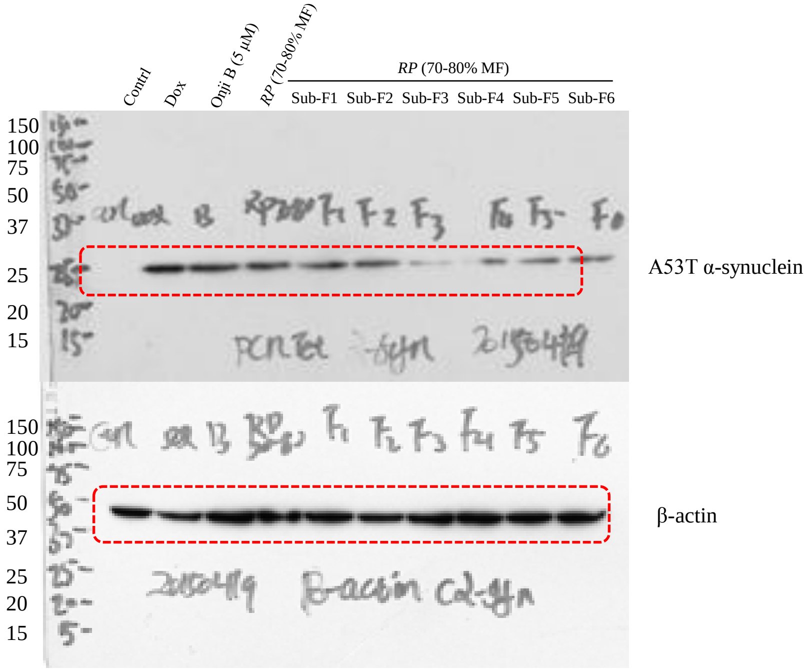

Supplementary Table 1: The characterization of the 17 chemical components of *RP* (70-80% MF) by using UHPLC-Q-TOF-MS analysis

Compounds	RT/ min	$[M-H]^-$ (m/z)	Major fragments ions (m/z)
Onjisaponin B (1)	14.381	1571.6888	1541.6781$[M-H-CH_2O]^-$
			1347.6394$[M-H-C_7H_{12}O_8]^-$
			1317.6215$[M-H-CH_2O-C_7H_{12}O8]^-$
			567.1914
			455.3146
			425.3064
Polygalasaponin	14.646	1673.7228	1643.7106$[M-H-CH_2O]^-$
XXXII (2)			1629.6971$[M-H-CO_2]^-$
			1541.6843$[M-H-Ara]^-$
			1449.668$[M-H-C_7C_{12}O_8]^-$
			1419.6065$[M-H-C_7C_{12}O_8-CH_2O]^-$
			1317.6243$[M-H-Ara-C_7C_{12}O_8]^-$
			993.3436$[M-680-H-]^-$
			861.3025$[M-680-H-Ara]^-$
			669.2231
			455.3153
			425.304

Onjisaponin L (3)	14.173	1847.7741	1817.7568[M-H-CH_2O]$^-$
			1703.7317[M-H-HMG]$^-$
			1673.7169[M-H-CH_2O-HMG]$^-$
			1479.6806[M-H-HMG-$C_7C_{12}O_8$]$^-$
			699.2333
			567.1918
			455.3149
			425.3045
Onjisaponin J (4)	14.921	1817.7634	1673.7202[M-H-HMG]$^-$
			1643.7199[M-H-HMG-CH_2O]$^-$
			1449.665[M-H-HMG-$C_7H_{12}O_8$]$^-$
			455.3158
			425.3046
Onjisaponin Vg /V(5)	14.679	1761.7363	1617.6936[M-H-HMG]$^-$
			1587.6814[M-H-HMG-CH_2O]$^-$
			1393.6445[M-H-HMG-$C_7H_{12}O_8$]$^-$
			455.3154
			425.3039

Supplementary Table 1 (con't)

Onjisaponin Fg (6)	15.185	1731.7282	1701.7148[M-H-CH_2O]$^-$
			1669.7259[M-H-Ara]$^-$
			1587.685[M-H-HMG]$^-$
			1557.6831[M-H-HMG-CH_2O]$^-$
			1363.6331[M-H-HMG-$C_7H_{12}O_8$]$^-$
			669.2237
			455.3154
			425.3045
Onjisaponin Ng (7)	14.701	1685.7207	1541.6775[M-H-HMG]$^-$
			1511.6801[M-H-HMG-CH_2O]$^-$
			1409.9753[M-H-Api-HMG]$^-$
			455.3191
			425.6806
Onjisaponin O (8)	14.713	1631.6997	1601.6859[M-H-CH_2O]$^-$
			1571.6641[M-H-2CH_2O]$^-$
			1407.6579[M-H-$C_7H_{12}O_8$]$^-$
			567.1898
			455.3162
			425.3044

Supplementary Table 1 (con't)

Onjisaponin Gg (9)	15.218	1599.6842	1569.6755[M-H-CH_2O]$^-$
			1455.6431[M-H-HMG]$^-$
			1375.5722[M-H-$C_7H_{12}O_8$]$^-$
			1231.5861[M-H-HMG-$C_7H_{12}O_8$]$^-$
			455.3158
			425.3051
Onjisaponin Y (10)	14.988	1409.6346	1379.6083[M-H-CH_2O]$^-$
			1185.5808[M-H-$C_7H_{12}O_8$]$^-$
			455.3153
			425.3043
Onjisaponin H (11)	14.252	1541.6801	1511.4853[M-H-CH_2O]$^-$
			1395.8726[M-H-Rha]$^-$
			1381.0504[M-H-MC]$^-$
			567.1902
			455.3155
			425.3037
Senegasaponin A (12)	14.769	1557.6733	1527.6675[M-H-CH_2O]$^-$
			1333.6182[M-H-$C_7H_{12}O_8$]$^-$
			1201.4548[M-H-$C_7H_{12}O_8$-Api]$^-$
			567.1976
			425.305

Supplementary Table 1 (con't)

Onjisaponin R (13)	13.62	1617.6954	1587.6847[M-H-CH_2O]$^-$
			1455.3985[M-H-Gal]$^-$
			1393.6403[M-H-$C_7H_{12}O_8$]$^-$
			1155.5581[M-H-$C_7H_{12}O_8$-TCA]$^-$
			455.3153
			425.3043
Onjisaponin F(14)	14.088	1587.6846	1557.6737[M-H-CH_2O]$^-$
			1377.6054[M-H-TMC]$^-$
			1363.63[M-H-$C_7H_{12}O_8$]$^-$
			1125.5491[M-H-$C_7H_{12}O_8$-TCA]$^-$
			455.3161
			425.3049
Senegasaponin B(15)	15.066	1425.6323	1395.6179[M-H-CH_2O]$^-$
			1201.5790[M-H-C_7H_{12}O8]$^-$
			567.1911
			455.3155
			425.3048

Supplementary Table 1 (con't)

Onjisaponin	14.836	1485.6529	1455.6416[M-H-CH_2O]$^-$
E (16)			1261.6003[M-H-$C_7H_{12}O_8$]$^-$
			675.3914
			567.1907
			455.3153
			425.3045
Onjisaponin	14.11	1703.7343	1673.7215[M-H-CH_2O]$^-$
A (17)			1479.6795[M-H-HMG]$^-$
			1449.6656[M-H-HMG-CH_2O]$^-$
			567.1922
			455.3156
			425.3045

Note: Rha = α-L-rhamnopyranosyl (146 Da); Gal = β-D-galactopyranosyl (132 Da); MC = (*E*)-4-methoxy cinnamoyl (160 Da); Api = β-D-apiofuranosyl (132 Da); Ara = β-D-arabopyranosyl (132 Da); HMG = 3S-3-hydroxy-3-methyl-5-pentanoic acid ester-H (144 Da); TMC = (*E*)-3,4,5-trimethoxy cinnamoyl; TCA = 4-methoxy cinnamoic acid (238 Da); CH_2O = (30 Da) $C_7H_{12}O_8$ = (224 Da)

Supplementary Table 2. The percentage of different type of saponins presented in the *RP* (70-80% MF), and *RP* (70-80% MF) sub-fractions (1-6)

Fractions (F) / Compounds	RP (70-80% MF)						
	+	F 1	F 2	F3	F4	F5	F6
Onjisaponin B	21.68			1.62	4.96	3.94	
Polygalasaponin XXXII	7.25				22.50	4.81	
Onjisaponin L	5.36	89.68	7.66				
Onjisaponin J	6.75		12.33	23.76	19.80		
Onjisaponin Vg/V	4.20		14.63	11.95	8.33		
Onjisaponin Fg	5.39			0.00	9.01	23.23	12.58
Onjisaponin Ng	1.31		6.00	5.18	3.24		
Onjisaponin O	12.29		16.52	37.95	4.85		
Onjisaponin Gg	1.19				1.06	4.18	4.24
Onjisaponin Y	1.84				0.00	1.67	10.24
Onjisaponin H	1.80				1.14		4.09
Senegasaponin A	0.64					5.60	
Onjisaponin R	8.84			1.92	20.69	2.36	0.00
Onjisaponin F	10.34			8.11	3.46	20.57	48.80
Senegasaponin B	1.38						17.39
Onjisaponin E	4.30				0.95	33.64	2.66
Onjisaponin A	5.46	10.32	42.87	9.51			
Total (%)	100	100	100	100	100	100	100

Supplementary Table 3. The distribution of different saponins (%) in all sub-fractions (1-6) of *RP* (70-80% MF)

Fractions / Compounds	*RP* (70-80% MF)-fractions						
	1	2	3	4	5	6	Total (%)
Onjisaponin B			16.81	33.35	49.84		100
Polygalasaponin XXXII				71.26	28.74		100
Onjisaponin L	94.16	5.84					100
Onjisaponin J		12.51	57.51	29.98			100
Onjisaponin Vg/V		26.13	50.88	22.99			100
Onjisaponin Fg				11.27	54.56	34.17	100
Onjisaponin Ng		25.69	52.86	21.45			100
Onjisaponin O		14.43	79.03	6.55			100
Onjisaponin Gg				5.84	43.32	50.84	100
Onjisaponin Y					12.33	87.67	100
Onjisaponin H				11.40		88.60	100
Senegasaponin A					100.00		100
Onjisaponin R			10.58	73.67	15.75		100
Onjisaponin F			7.80	2.16	24.05	65.99	100
Senegasaponin B						100.00	100
Onjisaponin E				1.36	90.37	8.27	100
Onjisaponin A	17.81	53.75	28.43				100
